# Transcriptional profiling of spiny lobster metamorphosis reveals three new additions to the nuclear receptor superfamily

**DOI:** 10.1186/s12864-019-5925-5

**Published:** 2019-06-28

**Authors:** Cameron J. Hyde, Quinn P. Fitzgibbon, Abigail Elizur, Gregory G. Smith, Tomer Ventura

**Affiliations:** 1 Genecology Research Centre, University of the Sunshine Coast, Sippy Downs, Queensland 4556 Australia; 2 Institute for Marine & Antarctic Studies (IMAS), University of Tasmania, Private Bag 49, Hobart, TAS 7001 Australia

## Abstract

**Background:**

The Crustacea are an evolutionarily diverse taxon which underpins marine food webs and contributes significantly to the global economy. However, our knowledge of crustacean endocrinology and development is far behind that of terrestrial arthropods. Here we present a unique insight into the molecular pathways coordinating crustacean metamorphosis, by reconciling nuclear receptor (NR) gene activity from a 12-stage, 3-replicate transcriptome in the ornate spiny lobster (Panulirus ornatus) during larval development.

**Results:**

We annotated 18 distinct nuclear receptor genes, including three novel NRs which are upregulated prior to metamorphosis and have hence been named the “molt-associated receptors” (MARs). We also demonstrate the ecdysone-responsive expression of several known molt-related NRs including ecdysone receptor, fushi-tarazu-F1 and E75. Phylogenetic analysis of the curated NR family confirmed gene annotations and suggested that the MARs are a recent addition to the crustacean superfamily, occurring across the Malacostraca from the Stomatopoda to the Decapoda. The ligand-binding domain of these receptors appears to be less conserved than that of typical group-1 NRs. Expression data from two other crustacean species was utilized to examine MAR expression. The Y-organ of the tropical land crab showed a decline in expression of all MARs from intermolt to post-molt. Tissue distributions showed gonad-enriched expression in the Eastern rock lobster and antennal gland-enriched expression in the tropical land crab, although expression was evident across most tissues.

**Conclusion:**

By mining transcriptome data, we have curated an extensive list of NR genes expressed during the metamorphic molts of P. ornatus, including three novel crustacean NRs which appear to play a role in the molting process. Divergence of the E-region of these new receptors indicates that they may have adopted a function that is unconventional for NRs. Based on expression patterns, we can confirm that a number of NRs play a role in the ecdysone cassette which regulates molting in crustaceans. This study describes in detail the molecular events surrounding crustacean molting and metamorphosis by taking advantage of the distinctive life history unique to achelatan crustaceans.

**Electronic supplementary material:**

The online version of this article (10.1186/s12864-019-5925-5) contains supplementary material, which is available to authorized users.

## Background

For over 500 million years, crustaceans have evolved to occupy virtually every corner of the world’s oceans [1], from abundant tropical reefs down to the deepest hadal trenches. Perhaps the key to the adaptive radiation of this clade was the evolutionary plasticity of the larval phase, which allowed new habitats to be rapidly explored through the adaptation of larval dispersal mechanisms. The result is a great variation in larval duration, size and morphology in the extant Crustacea, where multi-phasic lifecycles can see a single organism exploiting several distinct ecological niches throughout its lifetime. Each of these larval phases is transitioned by a metamorphosis, which typically occurs at a critical time in development and may be directed by a plethora of biotic and abiotic factors [2].

Our understanding of molecular endocrinology in arthropods is quite well developed, with model insects lending an abundance of resources and knowledge to the field. Studies into developmental pathways are particularly prolific, and subsequently the basis for insect metamorphosis has been well-examined over the past few decades. In light of this work, crustacean researchers have made every effort to draw analogous conclusions from their aquatic subjects. While many crustacean genes have been defined by conventional ortholog-hunting, their reconciliation with the endocrine pathways which they regulate is often dubious, and such inference is not always so insightful as one might optimistically expect.

Since their timing is often unpredictable, molting and metamorphosis can be particularly difficult to study in vivo, and sampling of individuals during these events presents quite a logistical challenge. Although there are some methods that have been developed to approximate time of molt in adult crustaceans [2, 3], similar opportunities to examine the larval phase are hard to come by. However, there is one group of decapod crustaceans which lends itself exceedingly well to this purpose at this particular point in time. During larval development, spiny lobsters undergo two distinct, transparent larval phases which are unique to the Achelata - the first is the flat, leaf-shaped phyllosoma and the second is the nektonic, shrimp-like puerulus. One notable hallmark of phyllosoma metamorphosis is the withdrawal of the hepatopancreas (known as gut-retraction) from the cephalic shield, a process which continues for 1–3 days prior to the metamorphic molt. Since phyllosoma larvae of this stage reach a size of 20-40 mm, gut retraction can be traced by eye with relative ease, providing a visible index of metamorphic progression [4]. This unique trait, together with a relatively predictable intermolt period (approximately 10 days in our species of study), makes spiny lobsters a potentially useful model for studying crustacean larvae.

Historically, the study of these profound larval phases has been precluded by the immense difficulty of either obtaining them from the wild or culturing them in the laboratory [5]. Serendipitously, however, the lifecycle of several spiny lobster species has recently been closed at the University of Tasmania’s aquaculture facility, operated by the Institute of Marine and Antarctic Studies (IMAS) [6, 7]. Under the conditions provided by this facility it has been possible to obtain and experiment with spiny lobsters at all phases of the lifecycle in a highly reproducible manner.

By taking advantage of this opportunity we have constructed a transcriptome profile spanning late larval development of the ornate spiny lobster *Panulirus ornatus*, with a temporal resolution that is difficult to achieve in most crustacean larvae. This data provides a valuable insight into developmental gene expression in the critical days before metamorphosis and, more generally, the transcriptomic progression that defines three distinct physiologies adopted by a single crustacean species.

It is well-recognised that molting and metamorphosis in arthropods is regulated by the steroid hormone ecdysone, which is released in cyclical pulses to stimulate molting events [8]. The molecular function of ecdysone is primarily mediated by its binding to the ecdysone receptor (ECR), a prominent member of the nuclear receptor (NR) superfamily [9].

**Fig. 1:**
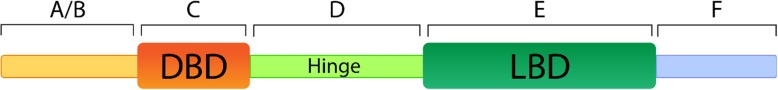
Nuclear receptor protein structure. The canonical nuclear receptor is defined by five regions which include two conserved domains, the DNA-binding domain (DBD) and the ligand-binding domain (LBD), separated by a disordered hinge region

The NRs are an ancient group of endocrine mediators which predate multicellular life [10]. They are subsequently highly conserved across Metazoa, and throughout evolution they have undergone duplication and neofunctionalization to form the large suite of receptors that we see in higher organisms today, which in humans amounts to 48 distinct receptors, including the thyroid hormone receptor and estrogen receptor [11]. The canonical NR is comprised of five distinct regions which incorporate two conserved domains – the DNA-binding domain (DBD) in the C region, and the ligand-binding domain (LBD) in the E region (Fig. 1). Together, these domains impart the conventional NR mechanism, with the DBD identifying and binding to specific response elements in the promoters of target genes, and the LBD forming an interactive dimer interface and ligand-binding pocket [12].

Following conventional steroid receptor function, the ECR forms a heterodimer with a second NR, the retinoid-X-receptor (RXR) [13], and this receptor complex can then directly bind DNA and modulate transcription of a specific suite of target genes, which includes many other members of the NR superfamily [14]. With a multitude of nuclear receptors and other transcription factors expressed differentially across tissues, the ecdysone signal is exponentially differentiated to coordinate the numerous physiological responses which orchestrate the molt, including chitin degradation, cuticle release and osmotic flux [12].

Due to their central role in the process of molting and metamorphosis, the focus of this study is to curate and observe the behaviour of the NR gene superfamily as larval development unfolds, with particular focus on the metamorphosis of the phyllosoma to the puerulus.

## Results

### Assembly and expression quantitation

Illumina sequencing produced a total of 1.89 billion raw reads, of which 96% remained as clean reads to give a mean output of 7.6 gigabases and GC content of 47% across samples. Trinity assembly produced a total of 289,397 transcripts across all samples (N50 = 1831 bp), with 28% of transcripts being annotated by at least one reference database (Additional file 1). A mean of 61% (SE ±1) of reads from each library were mapped back to 261,166 transcripts (90.3%) for expression quantitation, leaving 28,124 contigs to which no reads were mapped which were subsequently excluded from the dataset. Of these expressed transcripts, 28.7% (82,902) were successfully annotated by at least one database in the annotation pipeline, and 14.1% (42,158) were matched to predicted domains in the CDD database, 1978 of which would not have been annotated by the conventional pipeline alone.

**Fig. 2:**
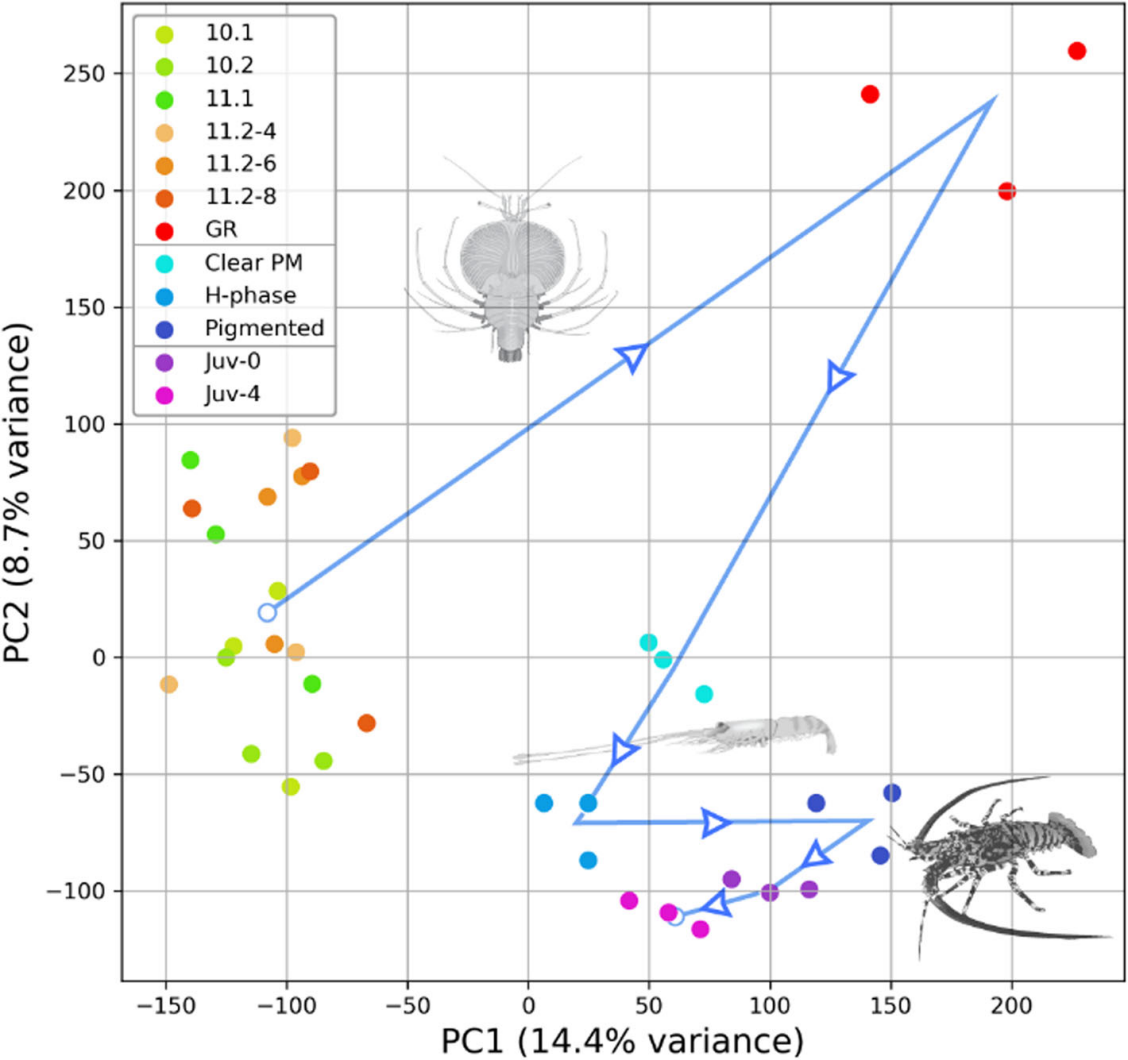
Principle component analysis of transcriptome expression data. Transcriptome expression data was filtered to include only transcripts which exceed 3 RLE (*n* = 96,534). In the legend, numerical labels (green and red) define the phyllosoma stages, which conclude with gut-retraction (GR) as phyllosoma metamorphosis is initiated. Three puerulus stages follow, and then finally two juvenile stages (Juv-*n*). Labels appended with -*n* denotes *n* days post-molt. The blue line connecting each cluster indicates the temporal sequence of stages, with blue arrows indicating direction

A subset of data was produced for PCA by filtering for e_max_ > 3, which explained 23% of variance with the two upper principle components (PCs). A two-dimensional plot of these PCs suggests that the phyllosoma stages preceding gut retraction share a similar transcriptomic profile, but each sampling point henceforth forms a distinct cluster whose distribution accurately reflects the temporal sequence (Fig. 2). This suggests relatively little transcriptomic change between phyllosoma stages 10.1 and 11.2-8d, followed by vast transcriptomic shift at the onset of gut-retraction. The puerulus, however, exhibits consistent transcriptomic shift between the three developmental points sampled. It is interesting to note that the only positive shift in PC1 is shown during pre-metamorphosis (i.e. GR phyllosoma and pigmented puerulus; Fig. 2), indicating that there are similarities in transcriptomic flux between the phyllosoma and puerulus pre-metamorphosis.

### Gene discovery

BLAST searching identified a total of 15 NR genes, comprising 81 variants. Amino acid/mRNA alignment indicated that some of these variants were the result of truncation or fragmentation during assembly. Consolidation of variants based on these observations resulted in a reformed total of 57 variants, including the ECR and RXR, as well as variants matching the recently discovered HR97 [ref] (Table 1). mRNA and amino acid alignments indicate that all variants are the result of alternative mRNA splicing (hereon referred to as isoforms), with the exception of the five HR97-like variants, which appear to be the product of four distinct genes, with one gene producing two isoforms (Table 1).

**Table 1:** *Panulirus ornatus* nuclear receptors

Gene	Variants	Splice region	AA	DBD	LBD
Po-E75	7	A/B E	707	X	X
Po-E78	2	A/B	429	X	-C
Po-ECR	5	D E	461	X	-C
Po-ERR	4	A/B	506	X	X
Po-Ftz-F1 α	4	A/B E	588	X	X
Po-Ftz-F1 β	1	–	453	X	X
Po-HNF4	2	E	553	X	X
Po-HR3	5	A/B	495	X	X
Po-HR38	2	A/B	695	X	X
Po-HR4	8	A/B	966	X	X
Po-HR78	3	CA/B	580	X	X
Po-HR96	1	–	654	X	X
Po-HR97/MAR	5	A/B	893	X	X
Po-RXR	6	A/BCE	452	X	X
Po-Svp	2	C/D	442	X	X

NR orthologs were identified in the transcriptome through BLAST searching, with alternative splicing sites based on predicted domain alignment. DBD and LBD presence in the longest open-reading frame encoded by each gene is indicated in the last two columns, with complete domain indicated by “X” and C-terminal truncated domain indicated by “-C”. Protein length (AA) corresponds to the longest open-reading frame

Reciprocal BLASTP search showed that the NRs identified have high homology to crustacean NRs in the NCBI database, with the exception of the HR97 variants. One Po-HR97-like matched to *D. magna* HR97b (accession AFJ97307.1) with an e-value of 2 × 10^− 120^, but the remaining Po-HR97s matched with e-values ranging from 10^− 35^ to 10^− 76^. We consider the latter to be a novel addition to the crustacean NR superfamily (as later results will show) and have provisionally named them the molt-associated receptors (MAR1-A, MAR1-B, MAR2, MAR3-A and MAR3-B, with -A and -B denoting isoforms) with NR superfamily designation of NR1Q, NR1R and NR1S, respectively. A full table of BLAST results for all NRs is available in Additional file 2.

Across the NRs identified in this study there is a high prevalence of splice variants, with only Ftz-F1β and HR96 having a single isoform (Table 1). Conversely, E75 and HR4 were shown to have seven and eight isoforms, respectively. The decapod ECR mRNA is known to be spliced in the A/B region, the hinge and the LBD [15], and here we show 5 variants that arise from splicing in both the hinge and LBD regions (Table 1).

To supplement phylogenetic analyses, orthologs for Po-HR97 and the three Po-MARs were obtained from existing transcriptome data (the eastern spiny lobster *Sagmariasus verreauxi* [4] and the tropical land crab *Gecarcinus lateralis* [16]) and from the NCBI’s Transcriptome Shotgun Archive (TSA) by tBLASTN search. An abundance of transcripts from these sources confirmed that the four distinct transcripts occur in various taxonomic lineages. Po-HR97 (with the highest homology to the *Daphnia* HR97 proteins [17]) orthologs occur across the Crustacea, being present in Branchiopoda (*D. magna* and *Triops newberrii*), Stomatopoda (*Oratosquilla oratoria*), and across the Decapoda. MAR1 and MAR2 were not present in the Branchiopoda or Maxillopoda but were present in the Stomatopoda (*O. oratoria*), Isopoda (*Proasellus meridianus* and *Bragasellus molinai*) and across the Decapoda, limiting their distribution to the Malacostraca. However, MAR3 orthologs were found only in Decapod and Euphausid (*Meganyctiphanes norvegica*) species, limiting the distribution of this transcript to the Eucarida. All of these orthologs were returned by BLAST matches with an E-value below 10^− 120^ and > 70% query cover. Domain prediction by RPS-BLAST suggests that all transcripts encode a DBD (E-value < 10^− 25^), with the exception of the *O. oratoria* HR97 transcript which appears to be truncated. LBDs were predicted in all transcripts with an E-value of below 10^− 15^ in all transcripts, with the exception of the MAR3 transcripts for which the E-value ranged from 10^− 3^ to 10^− 6^, suggesting that the LBD encoded by these transcripts has diverged considerably from the canonical structure. Accession numbers, BLAST scores, sequences and domain predictions for these transcripts have been made available in Additional file 2.

**Fig. 3:**
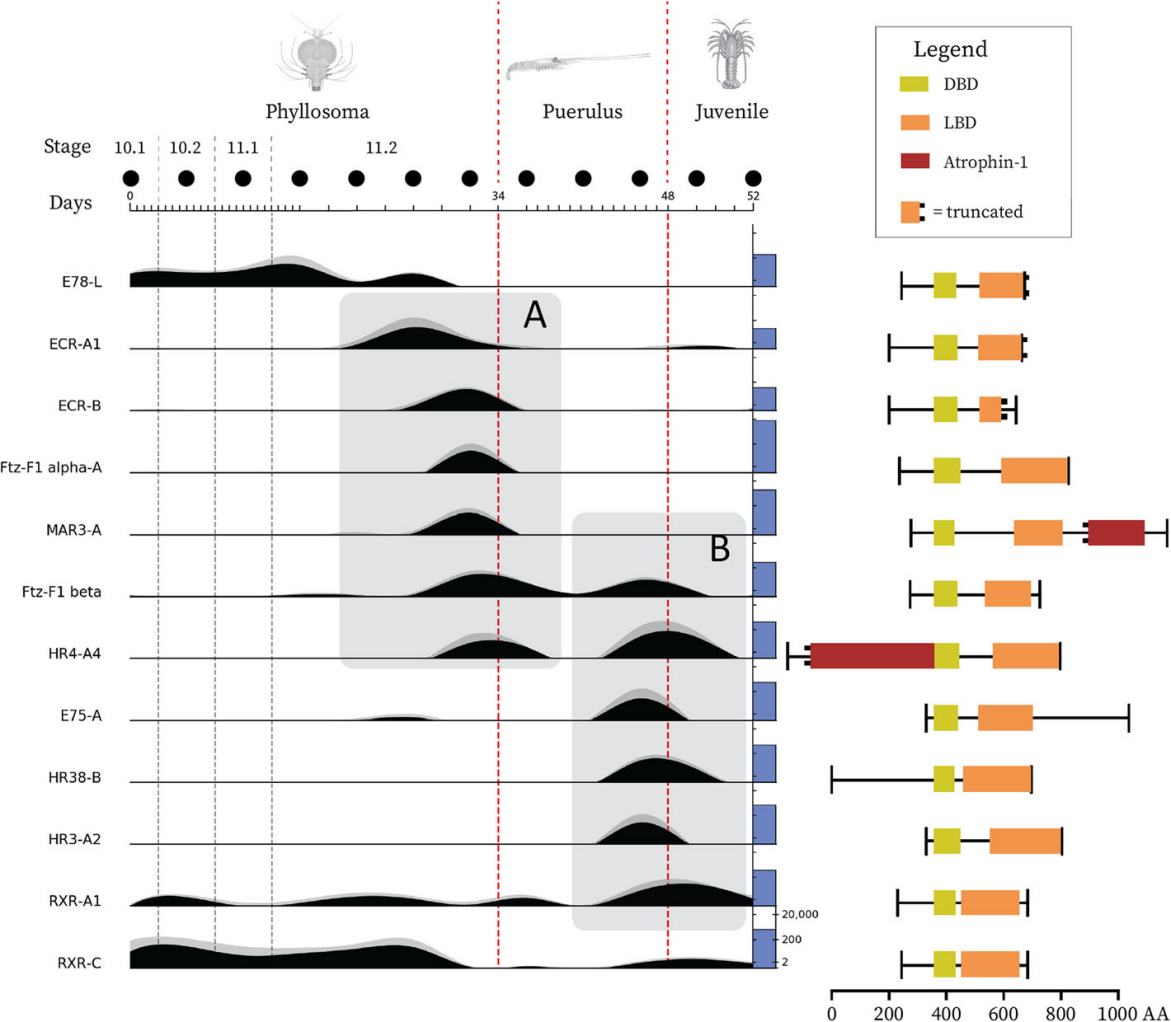
*Eriocheir sinensis* molt-associated receptor (MAR) exon-intron structure. mRNA sequences corresponding to the Es-MARs were aligned against genomic DNA to estimate the exon-intron structure. Exons are shown by green bars and introns by grey bars. The coding-DNA sequence (CDS) is shown below the exons with predicted DNA-binding domains (DBD) in red and ligand-binding domains (LBD) in purple, showing the relationship between splice junctions and protein sequence. The striped red bars upstream of the exons show a short length of sequence which did not align to the genome. All bars are drawn with sequence length scaled along the x-axis, with the exception of one intron for which the length is shown. Scaffold IDs correspond to the *E. sinensis* genome [18]

*E. sinensis* orthologs for MAR1, 2 and 3 were identified in the NCBI TSA archive (accessions GGQO01006193.1, GFBK01010337.1 and GGQO01007956.1, respectively) using a tBLASTN search with the *P. ornatus* MAR protein sequences as a query. Contigs matching these mRNA sequences were retrieved from the *E. sinensis* genome with exon identity of > 99.9% (Fig. 3). Genomic alignment showed that the Es-MAR mRNAs are composed of three exons, with the third exon consistently forming over 60% of the open reading frame (Fig. 3). Despite the DBD being highly conserved, this region is consistently found to be spanning at least one splice junction, while the more divergent LBD is consistently found in the middle of exon 3 (Fig. 3).

### Phylogenetic analysis

**Fig. 4:**
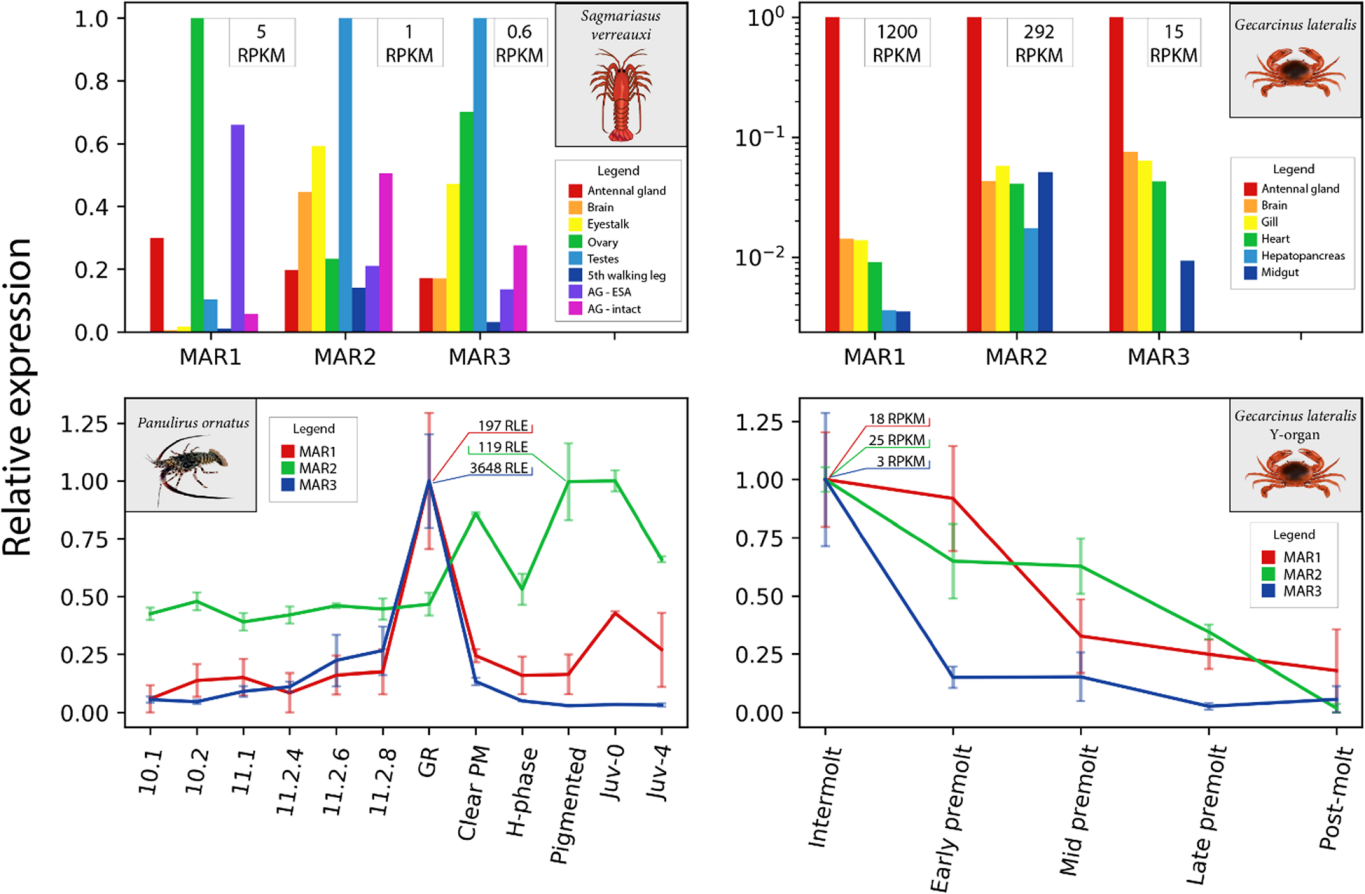
Phylogenetic reconstruction of *Panulirus ornatus* nuclear receptors, verified by the inclusion of respective orthologs from *Drosophila melanogaster* (Dr) and *Daphnia magna* (Da). Phylogenetic relationship was inferred using the maximum likelihood method based on the JTT matrix-based model [19], supported by bootstrap analysis with 500 replicates; the number adjacent to each node describes the percentage of trees in which the node’s sub-clade recurred. The scale bar shows amino acid substitutions per site. Designated NR families are shown on the outside of the phylogram with radial colors emphasising different genes

**Fig. 5:**
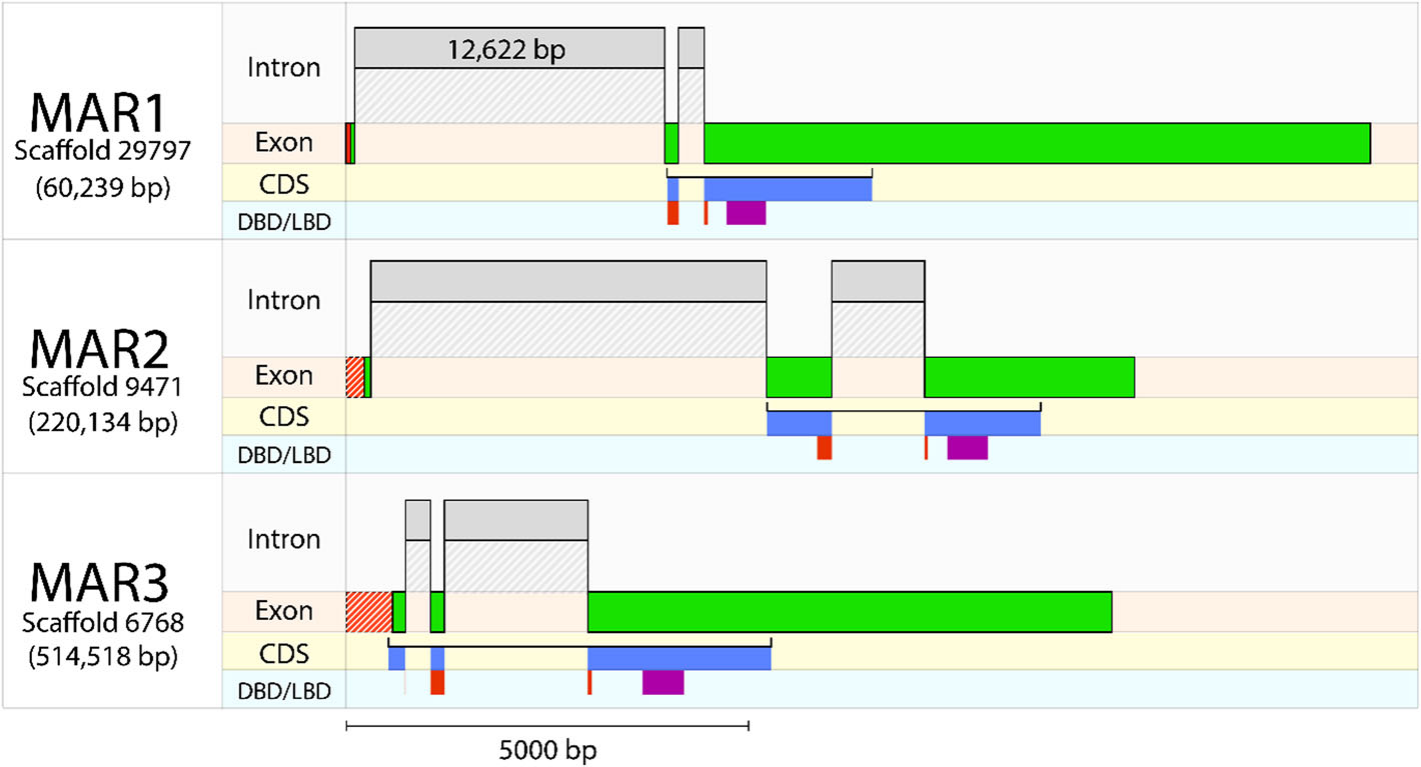
Phylogenetic relationship of *P. ornatus* nuclear receptors based on ligand-binding domains. Phylogenetic analysis was conducted with the maximum likelihood method based on the JTT matrix-based model, supported by bootstrap analysis with 500 replicates. Associated bootstrap values are shown beside each node. The scale bar shows substitutions per site and the chequered background highlights the novel NR genes identified in this study. *P. ornatus* branches are highlighted in bold. The six canonical NR families are represented by *P. ornatus* transcripts identified in this study, with the NR1 group being supported by the inclusion of a *Salmo salar* thyroxine receptor. Vitamin D receptor-like (VDR-like) sequences from the barnacle *Lepas anatifera* and the horseshoe crab *Limulus polyphemus* were used to root the phylogram. HR97-like sequences are presented for representatives across the Crustacea to demonstrate the relationship of the new NRs across their taxonomic range. Three novel HR97-like genes have been provisionally named the molt-associated receptors MAR1, MAR2 and MAR3 (with the family designations NR1S, NR1Q and NR1R, respectively)

Phylogenetic relationship of *P. ornatus* NRs based on the DBD amino acid sequences shows an identical superfamily structure to that described in *Drosophila melanogaster* [12], with the NRs clustering into six distinct families as conventionally described (Fig. 4). NR annotation was confirmed by consistent clustering of genes with their respective *Drosophila* and *Daphnia* orthologs. As previously shown in *Daphnia magna*, the HR97 gene forms a distinct clade within the NR1 group [20], and the MARs appear to be derived from within this lineage. To further examine the MARs, a second analysis of the LBD sequences was carried out, incorporating the crustacean HR97 and MAR orthologs identified earlier. Paraphyletic clustering of the HR97-like sequences resulted in only one of the identified variants falling within the previously identified Da-HR97 clade (Fig. 5). MAR1, MAR2 and MAR3 form a separate monophyletic clade with a greater distance to Dp-HR97 than the ECR, and the pairwise distance between each of these receptors at least equal to that between Po-ECR and Po-HR97 (Fig. 5). Phylogenetic inference suggests that divergence occurred sequentially throughout the evolution of crustaceans, with HR97 arising prior to the Pancrustacean ancestor, followed by the divergence of MAR1 and MAR2 in the early Malacostraca, as indicated by their presence in the Stomatopoda, but not the Branchiopoda and Maxillopoda. Finally, MAR3 is found only in the Eurycarida and is therefore likely to have diverged the most recently, although phylogenetic distance suggests that MAR3 is less conserved than MAR1 and MAR2 (Fig. 5). Combined with the high E-value of LBD predictions by RPS-BLAST, this indicates that the LBD of MAR3 has somewhat departed in structure from the canonical LBD defined in vertebrates.

### Gene expression analysis

Filtering of transcripts to e_max_ > 1 RLE produced a subset of expression data comprising 159,696 transcripts (61% of total transcripts). Of this subset, 69,037 (43%) were differentially expressed (DE; fold change> 4) between the intermolt (stage 11.2-4d) and gut-retracting phyllosoma; 6616 (9.6%) of the DE group were confirmed to be significant (SDE) based on an independent Welch’s T-test (*p* < 0.05) (Additional file 2). Out of the 76 transcripts identified as NRs by conserved domain prediction (i.e. transcripts containing a predicted DBD and LBD), 8 were present in the SDE group (Additional file 2). After being filtered for generic phrases, the fifth most frequent occurrence in the Nr annotations (after “kinase”, “mitochondrial”, “receptor” and “zinc finger”) was “cuticle” (76 occurrences), which likely relates to the epidermal detachment and remodelling which occurs during the metamorphosis. Other prominent annotations in this group were “JHE-like carboxylesterase” (8 occurrences) and “farnesoic acid O-methyltransferase” (4 occurrences), which perhaps indicates a role of the methyl farnesoate pathway during metamorphosis, as has been hypothesized for some time in crustaceans [21].

**Fig. 6:**
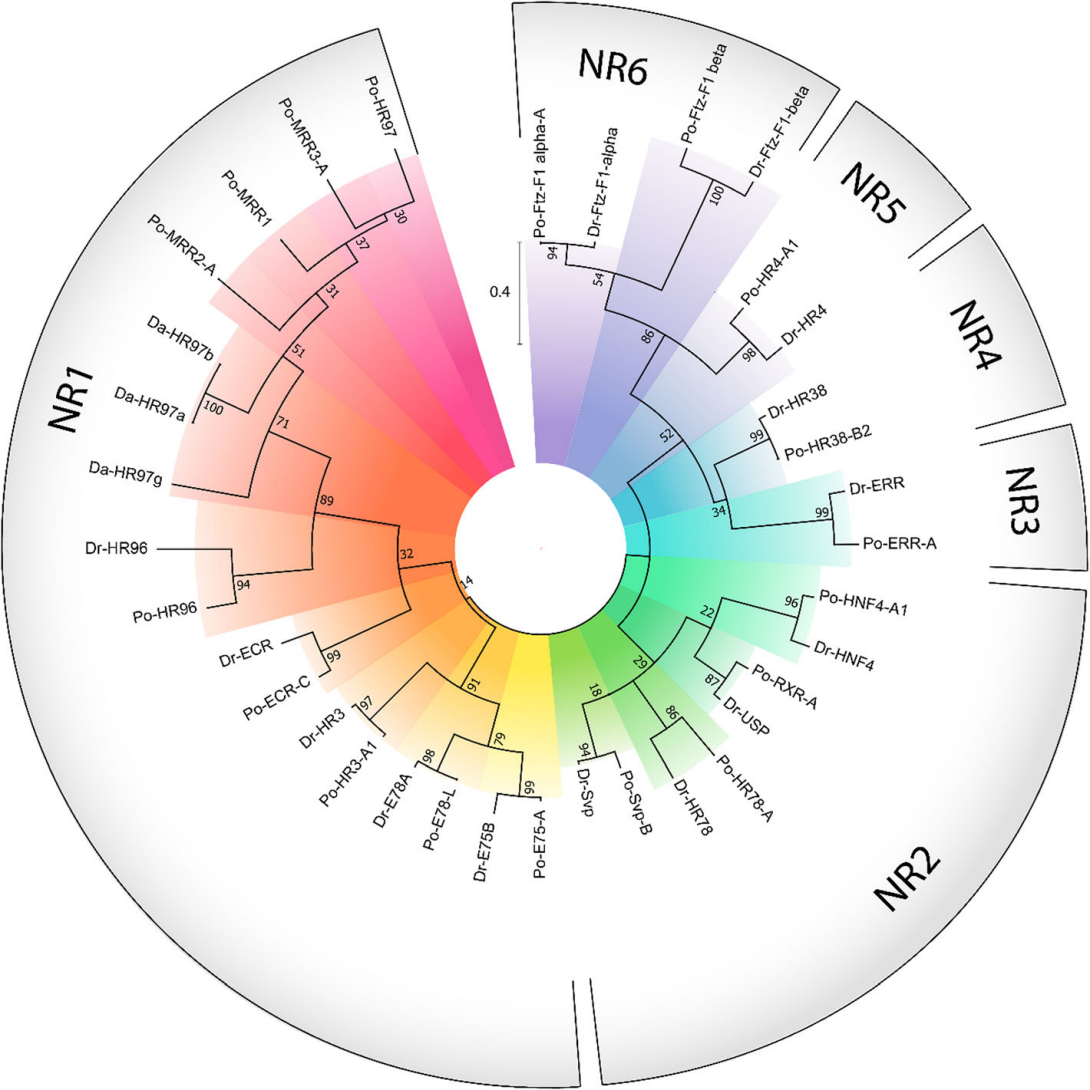
*P. ornatus* nuclear receptor gene expression and predicted domain structure Gene expression measured by RLE is plotted for 12 nuclear receptors throughout the 12 developmental stages sampled, covering three phyllosoma molts (grey dashed vertical lines) and the phyllosoma and puerulus metamorphoses (red dashed vertical lines). The scale bar above the expression plots shows time in days, drawing attention to the higher temporal resolution during the pre-metamorphic 11.2 stage. The solid black circles above this scale bar denote sampling events. Each level of the plot represents the relative expression of a nuclear receptor, measured as mean RLE (*n* = 3), normalized to the maximum expression of each gene (black area plots). The grey area plot stacked on top shows the standard error. The absolute expression level in RLE is shown as a log-scaled blue column on the right-hand edge of each expression plot; the first level includes a scale bar which applies to all levels. The corresponding protein domain structure is shown to the right of each expression plot level, as predicted by NCBI’s CD-search tool. Box A draws attention to a series of nuclear receptors which express prior to the phyllosoma metamorphosis and box B highlights those which express prior to the puerulus metamorphosis.

As indicated by the analysis presented above, the NRs show some interesting expression profiles across larval development, with some isoforms expressing specifically at either the phyllosoma or puerulus metamorphosis. Figure 6 presents the relative expression profiles of 12 NR transcripts which show stage-specific expression. Box A draws attention to a group of NRs (represented by Po-ECR, Po-Ftz-F1, Po-MAR3-A and Po-HR4) which peak in expression prior to phyllosoma metamorphosis. Likewise, box B draws attention to a group (represented by Po-Ftz-F1, Po-HR4, Po-E75, Po-HR38, Po-HR3 and Po-RXR) whose expression peaks in the pigmented puerulus, again prior to metamorphosis. Although there is considerable overlap in these groups (Po-Ftz-F1β and Po-HR4), some NRs certainly show a degree of specificity towards either phyllosoma or puerulus metamorphosis. Additionally, Po-E78-L is shown quite clearly to be phyllosoma-specific, declining in expression during the 11.2 stage before completely vanishing at gut-retraction (Fig. 6). Po-RXR-C shows a similar pattern of expression, disappearing during the puerulus phase before reappearing in the juvenile. Differential expression is evident between Po-RXR transcripts, as demonstrated by Po-RXR-A1 which peaks in expression around the puerulus post-molt. A full series of expression plots for all NR transcripts can be found in Additional file 3, and corresponding expression data in Additional file 4.

Taken together, the expression of ECR and E75 transcripts suggests that the pre-molt ecdysone pulse was reflected in the 11.2-8d phyllosoma and the pigmented puerulus transcriptomes, with E75 being less prominent in the phyllosoma metamorphosis. The highest expression of any NR isoform is shown by Ftz-F1 alpha-A during phyllosoma gut-retraction, where expression increases dramatically from 102 to 15,487 RLE (Fig. 6, Additional file 2), which likely indicates that this isoform is prominent across a wide distribution of tissues in the phyllosoma.

### Domain prediction

Annotations of NRs were confirmed by prediction of DBD and LBD by RPS-BLAST (Table 1), revealing some interesting observations of NR structure. Despite the consistent incorporation of a DBD and LBD region, the features surrounding these well-defined structures show a high degree of variability between receptors. Notably, the ECR isoforms which exhibit cascading expression peaks at phyllosoma pre-metamorphosis show structural differences in their LBDs, with a full-length LBD predicted for the earlier Po-ECR-A and a C-terminal truncated LBD for the later Po-ECR-B (Fig. 6).

More generally, the A/B region comprises around 200AA in the NR1 group NRs, whereas in Po-HR38 it forms nearly half of the protein sequence. In the Po-HR4-A transcripts the A/B region is highly extended and incorporates a predicted partial atrophin-1 domain, which also occurs in the F region of MAR3. Atrophins are an obscure group of proteins which are hypothesized to function as corepressors in transcription factor binding [22]. Although their function is poorly described, mutant alleles of atrophin-1 have been associated with cancer and neurodegenerative disease in humans [23]. The Pfam atrophin-1 domain is very large, spanning the entire 1175AA of the *Mus musculus* Atrophin-1 protein, which perhaps explains why only a fragment of the domain was matched to the NRs. The inclusion of an atrophin-1-like sequence in a NR protein has, to our knowledge, never been described.

Examination of MAR3 in nine decapod species indicates the F region is not well conserved, with Atrophin-1 domains being predicted in only three homarid lobsters and one crab. Interestingly, the four remaining transcripts were assigned a number of other predicted domains in place of atrophin-1, including EIF4E-T, Herpes_BLLF1, KAR9, Med15 and Med26_M (Additional file 4). Given the consistently low score (E-value > 10^− 5^), we believe that these matches could be explained by a conserved motif which is shared between these predicted domains. MAR1 and HR97 have both matched additional non-canonical domains, SSDP and PRK13729 (e-value 9 × 10^− 7^ and 5 × 10^− 3^), respectively, both of which are truncated at the C-terminus. Finally, the two MAR1 isoforms contain a predicted “Herpes ICP4 C superfamily” domain (e-value 9 × 10^− 3^) which again is truncated at the C-terminus. A full summary of NR domain predictions is available in Additional file 5.

### Expression of MAR orthologs across species

**Fig. 7:**
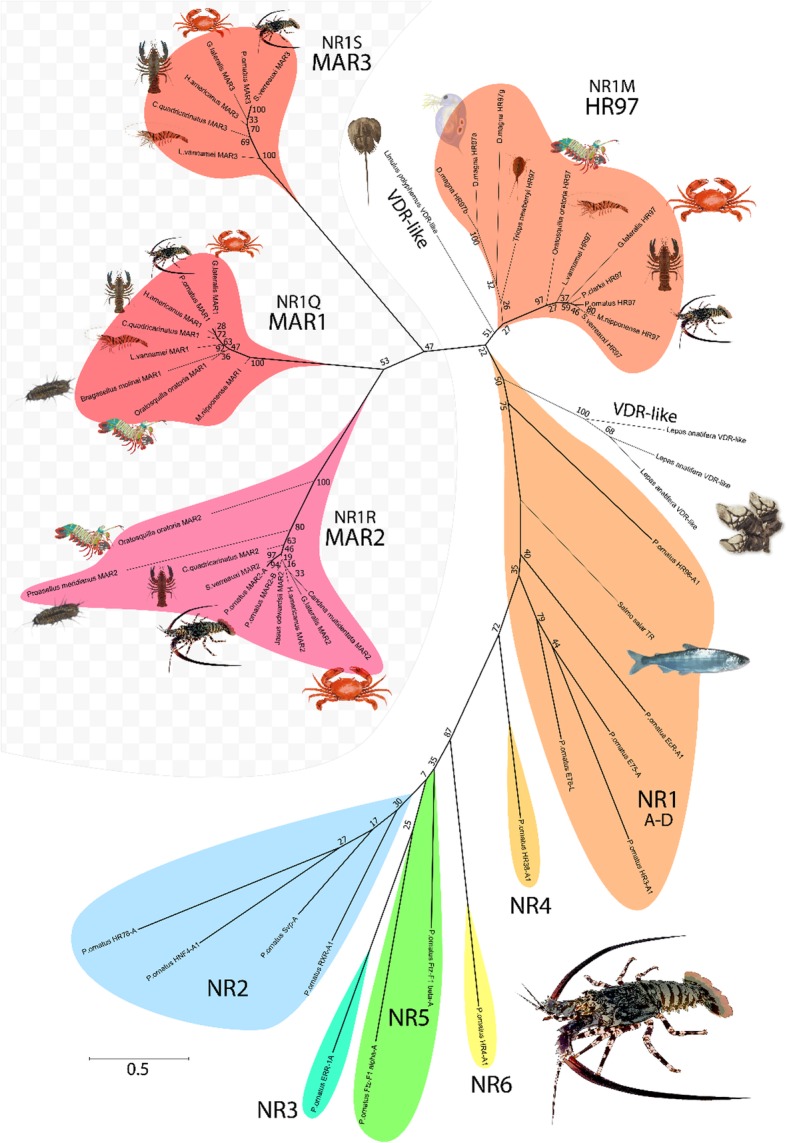
Comparison of molt-associated receptors’ (MAR) expression in three decapod species. Expression data for MAR orthologs was obtained from previous transcriptome studies. The top panels show the distribution of MAR expression across tissues for *S. verreauxi* [4] and *G. lateralis* (unpublished data). The bottom panels show temporal expression for *P. ornatus* on the left (this study) and the *G. lateralis* Y-organ throughout the molt cycle on the right [16]. All expression is shown in relative units with the absolute maximum expression of each transcript shown on the plot. Standard error is shown for the plots in the bottom panels (*n* = 3). Note the use of log scale in the y-axis of the top-right panel

Following the identification of MAR transcripts, we utilized data from previously described transcriptomes to investigate MAR expression in different species, including a tissue distribution from the Eastern spiny lobster *S. verreauxi* [24] and a tissue distribution (unpublished data) and Y-organ molting profile of the tropical land crab *G. lateralis* [16]. The MARs show moderate variation in expression patterns between species, with highest expression shown by the gonad in *S. verreauxi* and the antennal gland in *G. lateralis* (Fig. 7). Expression of the MARs in the *G. lateralis* Y-organ shows the opposite trend to that observed in *P. ornatus* larvae, with expression decreasing towards the molt (Fig. 7). Expression of the MARs during *S. verreauxi* phyllosoma metamorphosis ([4]; data not shown) correspond well with the expression patterns shown in this study in *P. ornatus* (Fig. 7).

## Discussion

Here we report a comprehensive list of NRs found in an achelatan crustacean, whose unusual biology we have exploited to gain a high-resolution profile of gene expression during larval development. The study has taken focus on key NRs of the ecdysone cassette, which is central to the regulation of arthropod molting and metamorphosis, and in doing so we have also exposed three novel crustacean NRs and traced their evolutionary history back to the early Malacostraca.

PCA of gene expression data suggest that the developmental stages examined were accurately represented by transcriptomic data, with the 12 developmental points sampled exhibiting seven distinct transcriptomic profiles (Fig. 2). Furthermore, transcripts which were significantly differentially expressed around phyllosoma metamorphosis included a number of annotations which are thought to associate with metamorphosis, including cuticle-related proteins and metabolic enzymes. Differential expression shown by NR-encoding transcripts reflects the well-established function of NRs as key developmental regulators, with many transcripts only appearing in close temporal proximity to metamorphosis (Fig. 6). Additionally, transcripts such as E75-C, E78-L and RXR-C (Fig. 6) which are more constitutively expressed but are confined to a particular larval phase, which indicates that they are either unique to phase-specific tissues or molecular processes. These expression patterns reflect previous findings in Drosophila, with E75-A and E75-C showing remarkably similar expression profiles between the two species (Additional file 3) [12], where E75-A exhibits ecdysone-induced peaks in mRNA levels and E75-C show a gradual increase in expression throughout larval development, culminating in a peak at head eversion in Drosophila and at puerulus pre-metamorphosis in *P. ornatus*. These similarities in expression suggest that the function of E75 isoforms is strongly conserved throughout the arthropods and also adds weight to the hypothesis that the puerulus and pupa phases are developmentally analogous.

Prior to phyllosoma metamorphosis, the successive appearance of various ECR isoforms as well as E75 and Ftz-F1 reflects well the conventional definition of the “ecdysone cascade”, the transcriptional response which follows ecdysone release [12]. This indicates that the timing of our sampling was sufficient to capture and represent this critical and elusive event and indicates that ecdysone release in the late phyllosoma of *P. ornatus* occurs approximately 2 days prior to the molt, in agreement with a previous study of this species [25]. Differential expression of ECR isoforms is concurrent with previous findings [15], with ECR-A and ECR-B peaking in expression prior to phyllosoma gut-retraction and ECR-C being more constitutively expressed (Fig. 6, Additional file 3). Phase-specificity of isoforms (E75, Ftz-F1, RXR, HR38 and HR78) suggests that differential splicing of NRs is important in distinguishing molecular regulation of the different larval phases. The structural distinction of such isoforms has already been indicated by a number of functional studies, with alternative-splicing of the LBD and hinge regions being associated with differential ligand and DNA binding affinity [14, 15, 26]. However, the absence of E74, TLL and HR39 was unexpected due to previous reports in other crustaceans [27, 28], and either indicates that these NRs are inactive during larval development or that they are absent in this particular clade of crustaceans.

While we have made every effort in this study to ensure validity and thoroughness, it is appropriate to address some limitations of our approach. The high temporal resolution shown in our study was gained at the cost of whole-organism sampling, which ignores tissue-specific expression. Therefore, genes with localized expression will not be accurately represented in this data. The primary aim of this study was to explore the expression patterns of the NRs around metamorphosis, and our data clearly demonstrates a dichotomous suite of NRs which are upregulated at either or both metamorphic events. However, our data does not describe the behaviour of these NRs in the context of a non-metamorphic molt and therefore, given that the crustacean metamorphosis is essentially an elaborate molt, we cannot differentiate between molt-related and metamorphosis-related gene expression. Conducting this comparison in the future would better allow us to identify the molecular basis for determining the nature of a molt to be metamorphic or otherwise.

Given the distinct phylogenetic relationship between the new HR97-like transcripts identified across the Malacostraca, we propose that these new genes represent a crustacean-specific lineage of NRs distinct from the HR97 gene defined by Thomson et al. [17], which likely arose from serial duplication of the ancestral HR97 during crustacean evolution. Conservation of the DBD strongly suggests that the MARs are derived from an ancient NR1 ancestor, but LBD divergence infers that strong positive selection has since occurred, particularly in MAR3 which, despite its later appearance in the Eucarida, is the more divergent of the three new NRs. Each MAR transcript aligned to three different scaffolds of the *E. sinensis* genome of up to 550Kbp in length (Fig. 3), thus there is little evidence for synteny of these genes. The MAR proteins show high divergence in their LBDs, despite their apparently recent divergence, which indicates that this region has undergone strong positive selection. Furthermore, the extension of the F region to incorporate a new (though not highly conserved) domain strongly indicates that these novel NRs have undergone some degree of neofunctionalization – a hypothesis which can be confirmed by functional analysis in the future. It is especially interesting that the same domain was predicted in the HR4 A/B region, since this indicates similarity in the structure or function of these regions. HR4 is known to regulate insect development through transcriptional repression of ecdysone-related genes [29], so perhaps MAR3 has been adopted as part of this mechanism. It would be interesting to explore the function of this domain for a link between HR4 and MAR3 in the future.

The MARs show quite some variation in expression between the three species examined, with the adult tissue distribution in *S. verreauxi* suggesting more constitutive expression than in the larvae, where expression seems to be metamorphosis-specific in MAR1 and MAR3 (Fig. 7). Conversely, the Y-organ of *G. lateralis* shows the opposite trend to the *P. ornatus* phyllosoma with regards to the molt cycle, as expression is highest at the intermolt and decreases towards the molt. While interpreting this analysis of gene expression it should be considered that we are comparing not only tissues and life stages, but also very distinct taxonomic groups. However, given the limited divergence in the LBD region between decapod species (Fig. 5), it is not unreasonable to assume that molecular function is conserved between species. While this comparison is unlikely to define a specific function or mechanism, it does allow us to speculate as to the specificity of MAR function.

With these limitations in mind, it could be hypothesized that the expression observed in the Y-organ of the crab (the opposite to that seen in *P. ornatus* larvae) reflects negative regulation of Y-organ tissues, with repression being released towards the molt. Expression in adult tissues suggests that the MARs adopt new regulatory roles in later life. Sexually dimorphic expression in the *S. verreauxi* gonad indicates that the MARs may play a role in regulating the reproductive cycle, with MAR1 showing highest expression in the ovary, while MAR2 and MAR3 express at a lower level in the testes. On the contrary, in *G. lateralis*, highest expression of the MARs consistently occurs in the antennal gland, with MAR1 showing especially high expression (Fig. 7). The antennal gland primarily functions as an excretory organ and plays an important role in osmoregulation, a process critical to the molting cycle [30]. The disparity in expression that we see between this crab and spiny lobster may indicate that the MARs have diverged in function between these distant lineages, but it could also reflect the fact that *G. lateralis*, a land-dwelling crustacean, has osmoregulatory requirements that are very different from that of a marine-dwelling spiny lobster.

## Conclusion

The finding of three novel NRs opens up some new questions. Which genes and processes do the MARs regulate? Has the divergent LBD retained any ligand-binding ability, or are they orphan receptors? The technology necessary to answer these questions is quite well-established and within the reach of most researchers. RNAi-mediated knock-down of the MARs would provide a phenotypic demonstration of the pathways that they regulate, with mRNA quantitation post-knockdown demonstrating more specifically the pathways that the MARs regulate. Further investigation of ligand-binding function could be explored by in silico 3D-modelling and ligand-docking [31] and confirmed with cell-based receptor assays [20].

While the discovery of novel NRs was unexpected, the aim of this study was to produce a time-series transcriptome which would elucidate the molecular happenings around metamorphosis in a representative crustacean. It has long been known that the signal to initiate molting is provided by the hormone ecdysone, and the data that we present shows in great detail a suite of genes which transmit and differentiate this hormonal signal. With each NR coordinating a different set of pathways and processes, their combined effect prepares the animal for the remarkable transformation that follows.

## Methods

### Larvae maintenance and collection

*Panulirus ornatus* larvae were cultured from wild-caught broodstock at IMAS, under proprietary culture conditions. Approximately 500 stage 9 and 10 phyllosoma were transferred from a mass rearing tank and distributed between two 110 L Kriesel tanks with flow-through open circulation. The water source was drawn from the ocean and treated by particle removal to 40 μm, foam fractionation, ozonation and carbon filtration before being heated to 28 °C and supplied to the culture tanks. Larvae were fed every 4 h with a proprietary formulated diet. Every morning, newly-molted individuals were transferred to isolated 5 L Kriesel tanks and their current stage and instar noted, in accordance with Smith et al. [32], who describe 11 distinct phyllosoma stages, each comprising between one and five instars. Given that the late phyllosoma had a molt period of approximately every 10 days, each individual could be accurately tracked and collected at a specific point in the molt cycle. Using this approach, animals were collected for RNA-extraction at 12 defined points throughout development. Harvested animals were immediately immersed in a saltwater ice slurry and staging was confirmed under a microscope. Larvae were then snap-frozen in liquid nitrogen and stored at − 80 °C. Phyllosoma were harvested at 4 days post-molt in stages 10.1, 10.2, 11.1 and 11.2 (where *n.x* denotes stage *n* instar *x*) - the latter of which culminates in metamorphosis [32]. In this final stage, additional samples were taken at 6 and 8 days post-molt, and at approximately 10 days when gut-retraction had commenced. The increased frequency of sampling at this stage provided a high-resolution profile of molecular activity as the animal prepares for metamorphosis. Further samples were taken from the post-molt, H-phase and pigmented puerulus (as described by Lemmens [33]) and then in the juvenile phase at zero and 4 days post-molt.

### RNA extraction

Whole larvae were taken from − 80 °C and mechanically homogenized in RNAzol® RT (Molecular Research Center) supplemented with 1% v/v β–mercaptoethanol. RNA was then extracted following manufacturer guidelines to yield approximately 500–3000 ng μl^− 1^ depending on developmental stage. Purity was assessed by spectrophotometer (Nanodrop 2100) and samples which fell below the manufacturer’s rejection criteria were re-precipitated with lithium chloride and cleaned by ethanol washing to improve RNA purity. RNA integrity was then evaluated with chip electrophoresis (Agilent Bioanalyzer) to ensure that samples were not degraded.

### High-throughput sequencing

Three RNA samples from each sampling point were prepared for shipping with RNAstable® LD (Biomatrica), with 2 μg of each sample being mixed with the reagent and air-dried overnight in a desiccator under negative pressure. The samples were sent to Novogene (Beijing) for paired-end sequencing in Illumina HiSeq™ 2500. RNA libraries were constructed by oligo(dT) mRNA enrichment, followed by random fragmentation and cDNA synthesis with random hexamers. This was followed by second-strand synthesis by nick translation and purification with AMPure XP beads. cDNA libraries were then finalized by further quality control, ligation of sequencing adaptors and PCR enrichment before sequencing was carried out.

### Transcriptome assembly & verification

Raw reads were trimmed to remove adapter contamination and reads with high uncertainty (*N* > 10%) or low base quality were discarded. Due to the absence of a reference genome, clean reads were de novo assembled using Trinity software [34] with hierarchical clustering by Corset [35]. Functional annotation was then inferred from seven databases including Nr, Pfam, KOG and Swiss-Prot (Additional file 1). Transcript expression quantitation was calculated by RSEM [36], producing raw read counts which were then used to calculate RLE (Relative Log Expression) with the software DESeq2 [37] on the Galaxy public web server [38]. A detailed summary of the software and parameters used in the assembly pipeline can be found in Additional file 1.

To examine expression data, transcripts were filtered based on the maximum mean RLE reached across samples (henceforth referred to as e_max_). With a threshold e_max_ of 3 RLE applied, the resulting subset of data were subjected to a principle component analysis (PCA) to visualize the variability in gene expression within and between developmental stages. This analysis was carried out in Python 3.6.5 with the pandas and scitkit-learn libraries, and the output was plotted using the matplotlib library [39–41].

### Gene discovery & sequence analysis

With over 280,000 transcripts and an inconclusive list of candidate genes suggested by the literature, a combination of approaches was used to examine the transcriptome data in order to persuade genes of interest to surface. With a 2-day resolution of gene expression around the phyllosoma metamorphosis, some emphasis was placed on short-listing transcripts which showed differential expression during this time, while also exhibiting the protein structure of a nuclear receptor or transcription factor.

Protein sequences were obtained for use as BLAST queries by searching the NCBI database [42]. Queries were selected by closest taxonomic relation to *P. ornatus*, excluding predicted sequences. Query sequences were obtained by searching the NCBI database for protein homologs for genes of interest, which were then used for probing the transcriptome by tBLASTn in BioEdit [43]. Corresponding homologs were extracted and translated to protein sequences using the BioPython module in Python 3.5.2 [44]}. Translated protein sequences were then confirmed by reciprocal BLASTP search of the NCBI nr database. Conserved domains were then predicted for the entire proteome (translated with BioPython and CDS prediction by longest predicted open reading frame) to confirm expected protein structures and allow structural comparison between genes and isoforms. Generating this data also allowed the searching and filtering of transcripts by predicted domain. Domain predictions were made with a local standalone build of the NCBI Conserved Domain search tool (CD-search - www.ncbi.nlm.nih.gov/Structure/cdd/wrpsb.cgi) [45]. This software utilizes a RPS-BLAST (Reverse Position-Specific BLAST) to match conserved domains from the CDD database, which includes NCBI-curated domains in addition to Pfam, SMART, COG, PRK and TIGRFAMs databases. The E-value threshold for reporting predicted domains was set to the default of 0.01. Conserved domain alignment data were processed using the rpsbproc tool (part of the local CD-Search package available at ftp://ftp.ncbi.nih.gov/pub/mmdb/cdd), the output from which was parsed in Python 3.6.5 and then plotted using the matplotlib library [41] to visualize the predicted domain structure encoded by each transcript.

Exon-intron structure was analysed for several genes by aligning *Eriocheir sinensis* cDNA against genomic contigs [18]. Sequences identified in the NCBI TSA archive were used as BLASTN queries to identify matching genome contigs, which they were then aligned against using NCBI’s online Splign tool [46] (www.ncbi.nlm.nih.gov/sutils/splign). The alignment coordinates generated by Splign were then used to plot exon-intron structure alongside the open reading frame and predicted domains.

### Gene expression analysis

Given that our approach sought to curate a specific family of genes from the transcriptome, we did not see the need for a statistical method to identify genes of interest. Expression plots were generated based on RLE and are displayed as relative expression (i.e. relative to the highest RLE reached by that transcript) and standard error was shown as a measure of significance, where replicates existed. To further contextualize the function of the MAR genes, additional transcriptome data were drawn upon - from the Eastern spiny lobster *Sagmariasus verreaxi* [24] and tropical land crab *Gecarcinus lateralis* [16]. The transcriptome profile from *S. verreauxi* shows gene expression across the adult tissues, while data from *G. lateralis* describes expression in the Y-organ across the molt cycle [16] and across an adult tissue distribution (unpublished data), with the latter being collected, processed and assembled with the same methodology as the former.

### Phylogenetic analysis

Annotation of the curated list of NR superfamily members was further validated by phylogenetic inference, with the hypothesis that the phylogenetic relationship observed in the insect NRs [12] would be conserved in *P. ornatus*. The phylogeny was based on the highly conserved DBD region, which was extracted from the translated mRNA sequences based on conserved domain coordinates predicted by CD-search. Amino acid sequences were aligned by Muscle [47] in MEGA 7 [48] with default parameters, followed by phylogenetic analysis by the maximum-likelihood method with 500 bootstrap replicates. The same procedure was followed to extract and analyse the LBD sequences to investigate new genes.

## Additional files

Additional file 1: “Sequencing report” - a spreadsheet with details of the sequencing report and assembly pipeline. Read mapping graphs are also shown to validate expression quantitation. (XLSX 823 kb)

Additional file 2: “Transcript log” – a spreadsheet containing gene expression, differential expression, annotation and predicted domain data. (XLSX 29369 kb)

Additional file 3: “Expression plots” – a series of gene expression plots for all identified nuclear receptors in the style of Fig. 6. (PDF 941 kb)

Additional file 4: “MAR transcripts” – A spreadsheet of sequences and predicted domain data used in the cross-species phylogeny of the MAR proteins. (XLSX 140 kb)

Additional file 5: “Nuclear receptor predicted domains” – A spreadsheet of the predicted domain data, including peptide coordinates and match statistics for all nuclear receptors reported in the study. (XLSX 59 kb)

## Data Availability

The datasets supporting the conclusions of this article are included within the article and its additional files, including transcript nucleotide and protein sequences, annotations, expression data and predicted domain data.

## Abbreviations

BLAST: Basic local alignment search tool
DBD: DNA-binding domain
ECR: Ecdysone receptor
e_max_: Maximum mean expression level of a transcript
GR: Gut-retraction
LBD: Ligand-binding domain
LRE: Fragments-per kilobase of transcript per million sequencing reads
MAR: Molt-associated receptor
NCBI: National Center for Biotechnology Information
NR: Nuclear receptor
PCA: Principle-component analysis
RNA-seq: Total RNA sequencing
RXR: Retinoid-X receptor
