# Correction to: Transcriptional profiling of spiny lobster metamorphosis reveals three new additions to the nuclear receptor superfamily

**DOI:** 10.1186/s12864-019-5990-9

**Published:** 2019-07-31

**Authors:** Cameron J. Hyde, Quinn P. Fitzgibbon, Abigail Elizur, Gregory G. Smith, Tomer Ventura

**Affiliations:** 1 Genecology Research Centre, University of the Sunshine Coast, Sippy Downs, Queensland 4556 Australia; 2 Institute for Marine & Antarctic Studies (IMAS), University of Tasmania, Private Bag 49, Hobart, TAS 7001 Australia

**Correction to: Hyde et al. BMC Genomics (2019) 20:531**

**https://doi.org/10.1186/s12864-019-5925-5**

Following publication of the original article [1] the authors noted an error in the order of the Figs. 3, 4, 5, 6, 7, which caused the figures and their legends to mismatch. The figure legends are correct, but the wrong images have been matched to them.

**Fig. 3:**
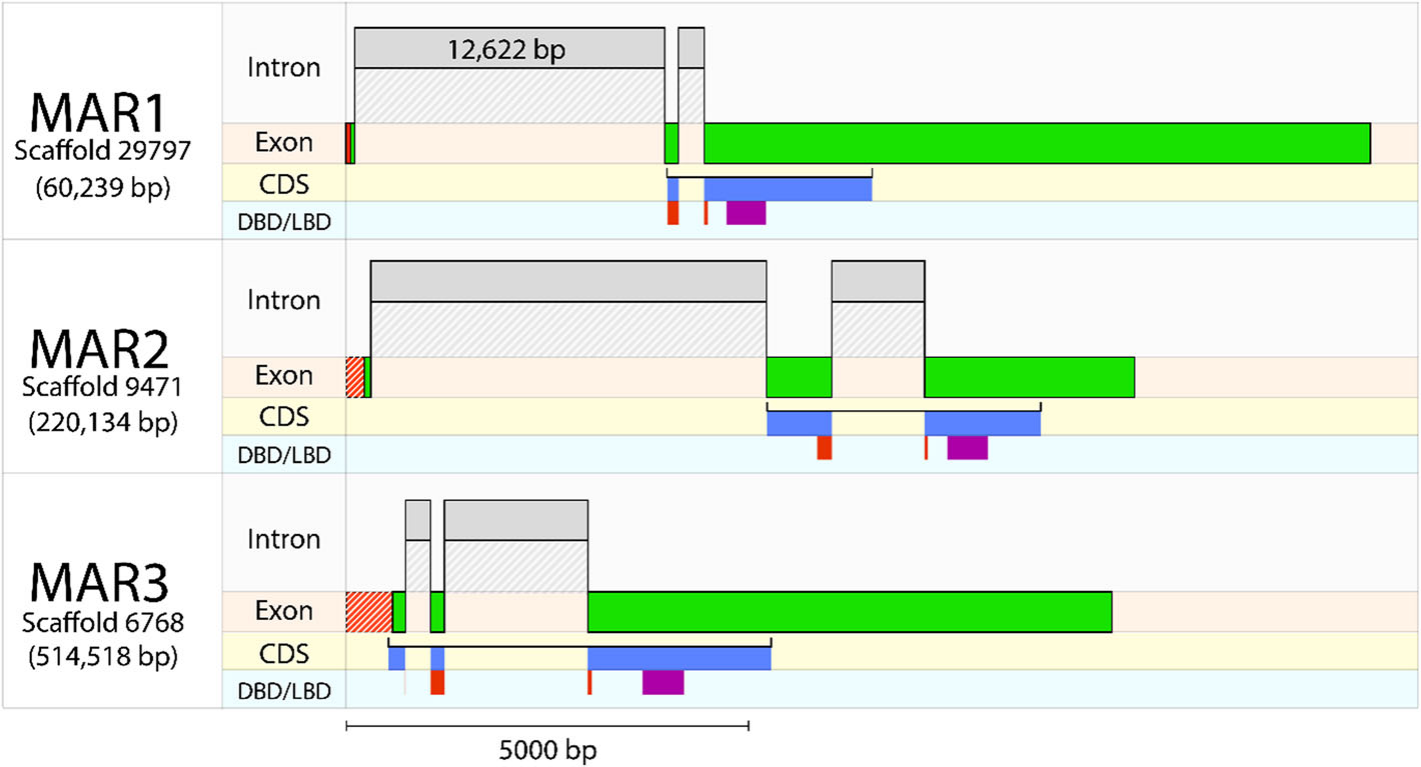
*Eriocheir sinensis* molt-associated receptor (MAR) exon-intron structure. mRNA sequences corresponding to the Es-MARs were aligned against genomic DNA to estimate the exon-intron structure. Exons are shown by green bars and introns by grey bars. The coding-DNA sequence (CDS) is shown below the exons with predicted DNA-binding domains (DBD) in red and ligand-binding domains (LBD) in purple, showing the relationship between splice junctions and protein sequence. The striped red bars upstream of the exons show a short length of sequence which did not align to the genome. All bars are drawn with sequence length scaled along the x-axis, with the exception of one intron for which the length is shown. Scaffold IDs correspond to the *E. sinensis* genome [18]

**Fig. 4:**
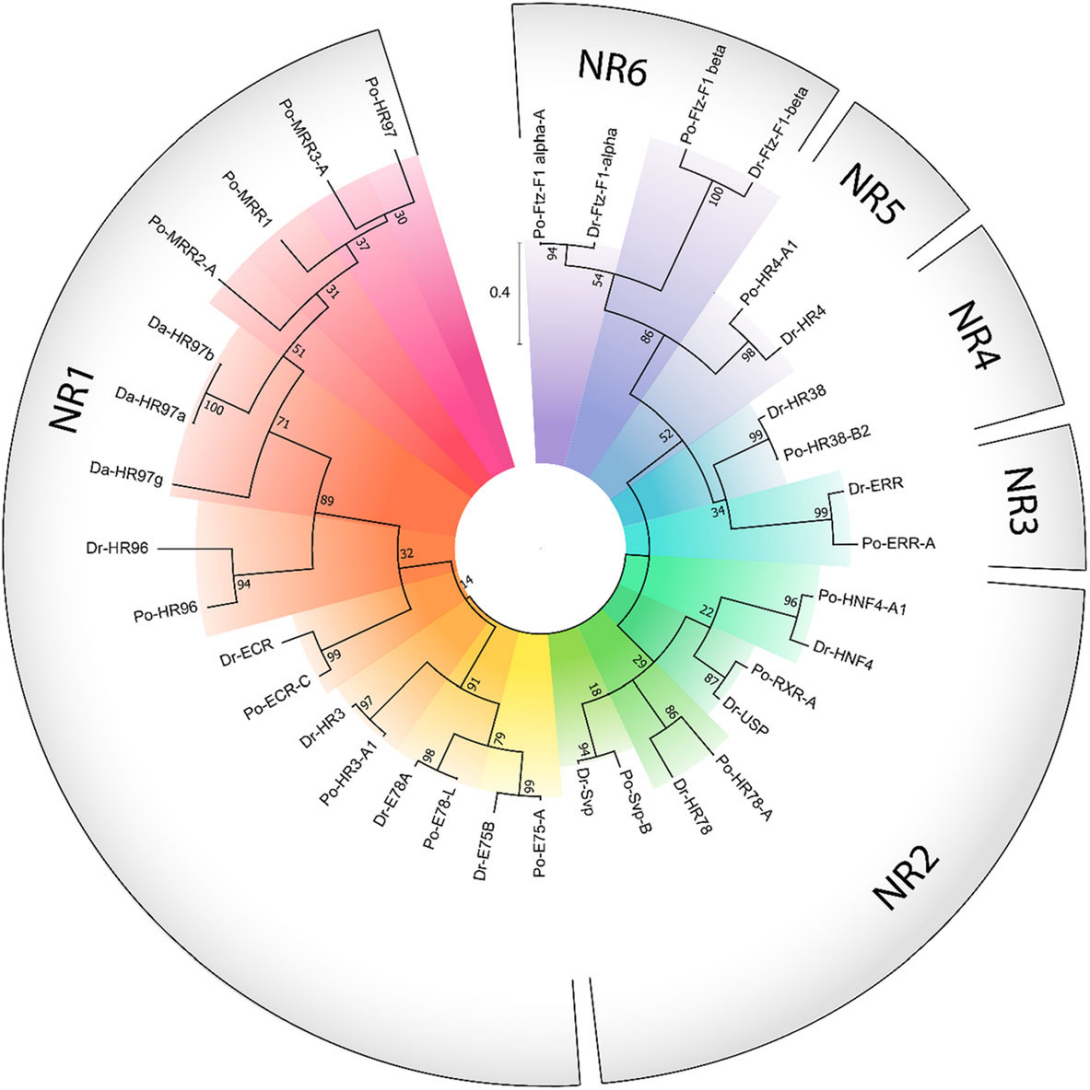
Phylogenetic reconstruction of *Panulirus ornatus* nuclear receptors, verified by the inclusion of respective orthologs from *Drosophila melanogaster* (Dr) and *Daphnia magna* (Da). Phylogenetic relationship was inferred using the maximum likelihood method based on the JTT matrix-based model [19], supported by bootstrap analysis with 500 replicates; the number adjacent to each node describes the percentage of trees in which the node’s sub-clade recurred. The scale bar shows amino acid substitutions per site. Designated NR families are shown on the outside of the phylogram with radial colors emphasising different genes

**Fig. 5:**
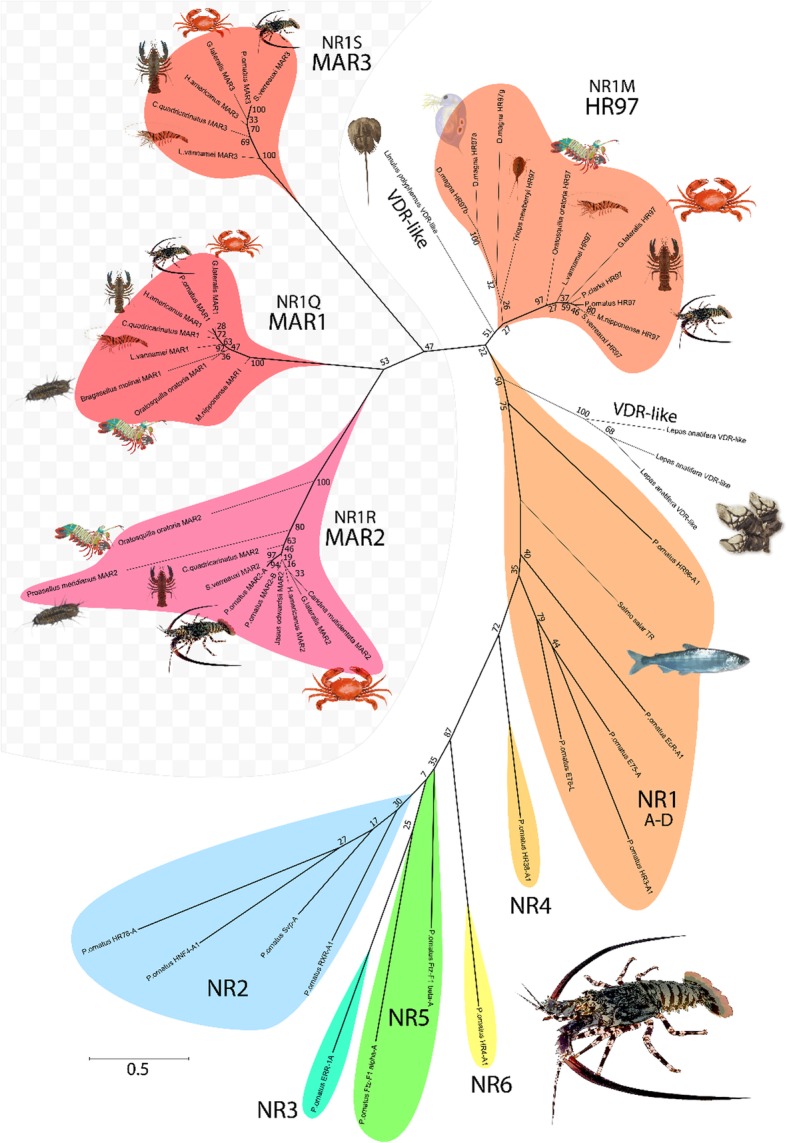
Phylogenetic relationship of *P. ornatus* nuclear receptors based on ligand-binding domains. Phylogenetic analysis was conducted with the maximum likelihood method based on the JTT matrix-based model, supported by bootstrap analysis with 500 replicates. Associated bootstrap values are shown beside each node. The scale bar shows substitutions per site and the chequered background highlights the novel NR genes identified in this study. *P. ornatus* branches are highlighted in bold. The six canonical NR families are represented by *P. ornatus* transcripts identified in this study, with the NR1 group being supported by the inclusion of a *Salmo salar* thyroxine receptor. Vitamin D receptor-like (VDR-like) sequences from the barnacle *Lepas anatifera* and the horseshoe crab *Limulus polyphemus* were used to root the phylogram. HR97-like sequences are presented for representatives across the Crustacea to demonstrate the relationship of the new NRs across their taxonomic range. Three novel HR97-like genes have been provisionally named the molt-associated receptors MAR1, MAR2 and MAR3 (with the family designations NR1S, NR1Q and NR1R, respectively)

**Fig. 6:**
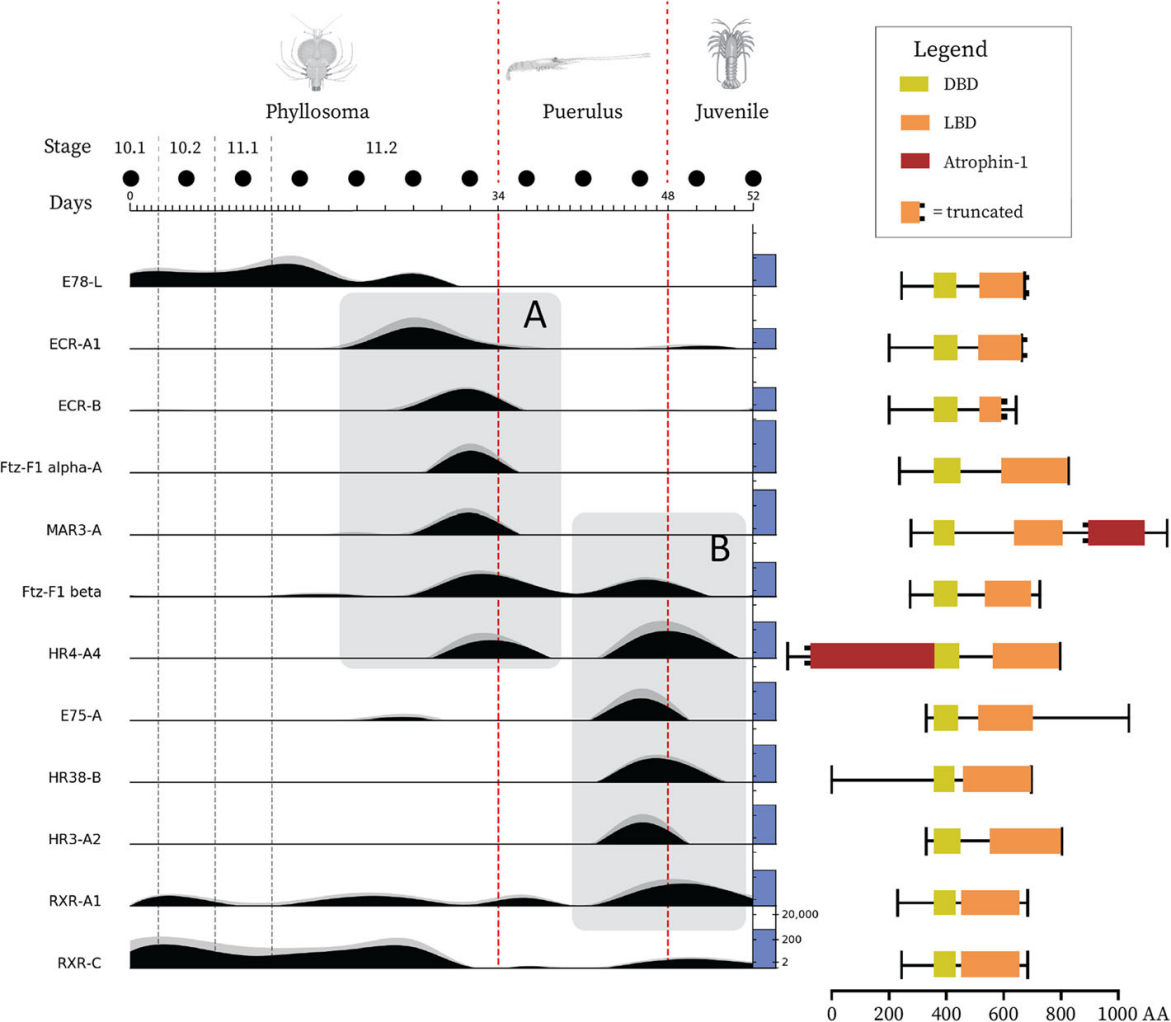
*P. ornatus* nuclear receptor gene expression and predicted domain structure Gene expression measured by RLE is plotted for 12 nuclear receptors throughout the 12 developmental stages sampled, covering three phyllosoma molts (grey dashed vertical lines) and the phyllosoma and puerulus metamorphoses (red dashed vertical lines). The scale bar above the expression plots shows time in days, drawing attention to the higher temporal resolution during the pre-metamorphic 11.2 stage. The solid black circles above this scale bar denote sampling events. Each level of the plot represents the relative expression of a nuclear receptor, measured as mean RLE (*n* = 3), normalized to the maximum expression of each gene (black area plots). The grey area plot stacked on top shows the standard error. The absolute expression level in RLE is shown as a log-scaled blue column on the right-hand edge of each expression plot; the first level includes a scale bar which applies to all levels. The corresponding protein domain structure is shown to the right of each expression plot level, as predicted by NCBI’s CD-search tool. Box A draws attention to a series of nuclear receptors which express prior to the phyllosoma metamorphosis and box B highlights those which express prior to the puerulus metamorphosis.

**Fig. 7:**
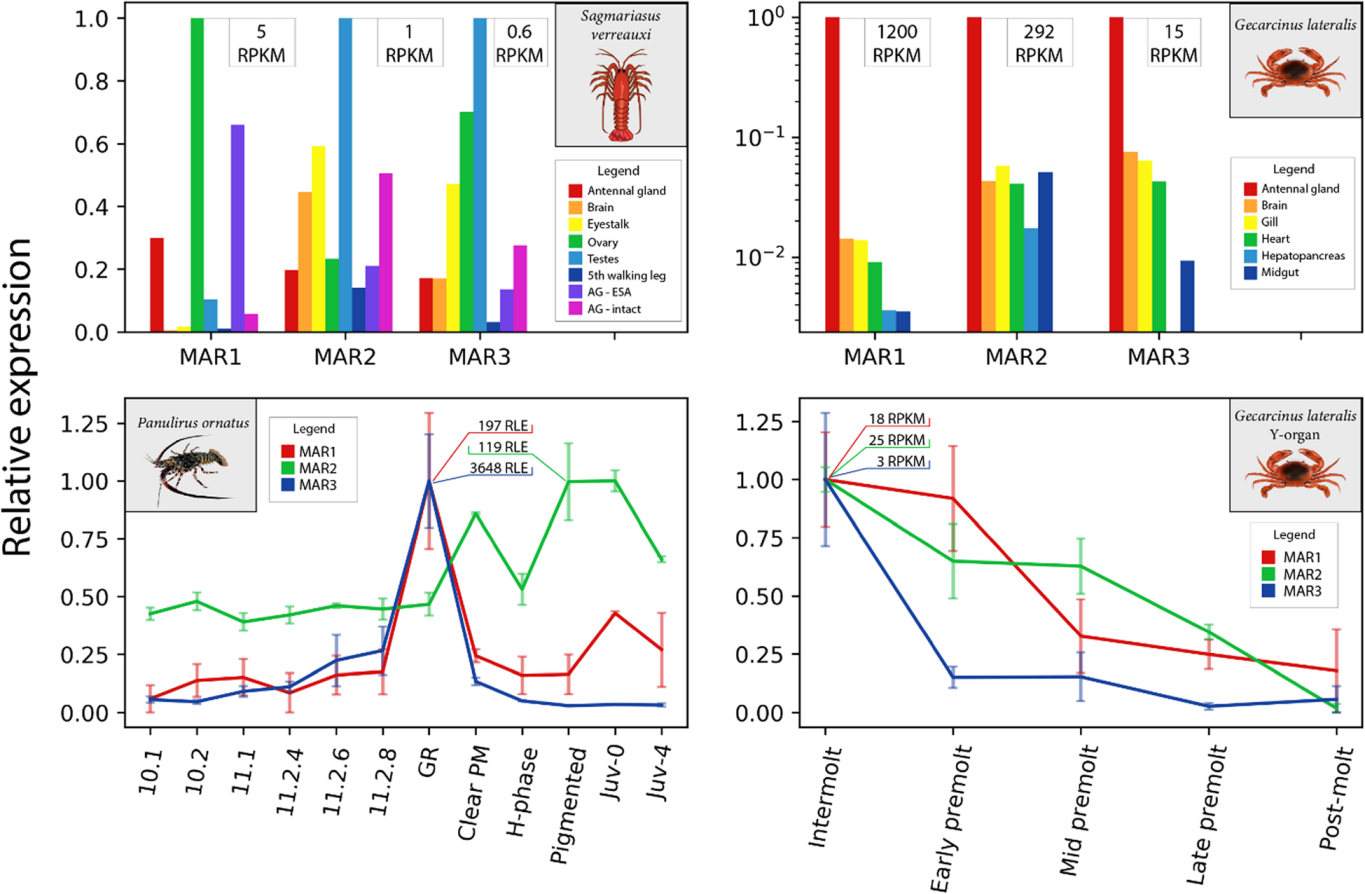
Comparison of molt-associated receptors’ (MAR) expression in three decapod species. Expression data for MAR orthologs was obtained from previous transcriptome studies. The top panels show the distribution of MAR expression across tissues for *S. verreauxi* [4] and *G. lateralis* (unpublished data). The bottom panels show temporal expression for *P. ornatus* on the left (this study) and the *G. lateralis* Y-organ throughout the molt cycle on the right [16]. All expression is shown in relative units with the absolute maximum expression of each transcript shown on the plot. Standard error is shown for the plots in the bottom panels (*n* = 3). Note the use of log scale in the y-axis of the top-right panel

The correct images with legends are given in this Correction article.
