# Supplementary material for: Transcriptional profiling of spiny lobster metamorphosis reveals three new additions to the nuclear receptor superfamily
Source: BMC Genomics. 2019 Jun 28;20:531. doi: 10.1186/s12864-019-5925-5 (PMC6599367; doi:10.1186/s12864-019-5925-5)
Supplement: Supplementary file 3 — “Expression plots” – a series of gene expression plots for all identified nuclear receptors in the style of Fig. 6. (PDF 941 kb) [file 12864_2019_5925_MOESM3_ESM.pdf]

Panulirus ornatus nuclear receptor expression

Supplementary figure 3: Whole-organism relative expression (RLE) of nuclear receptor genes across 12 points of larval development.

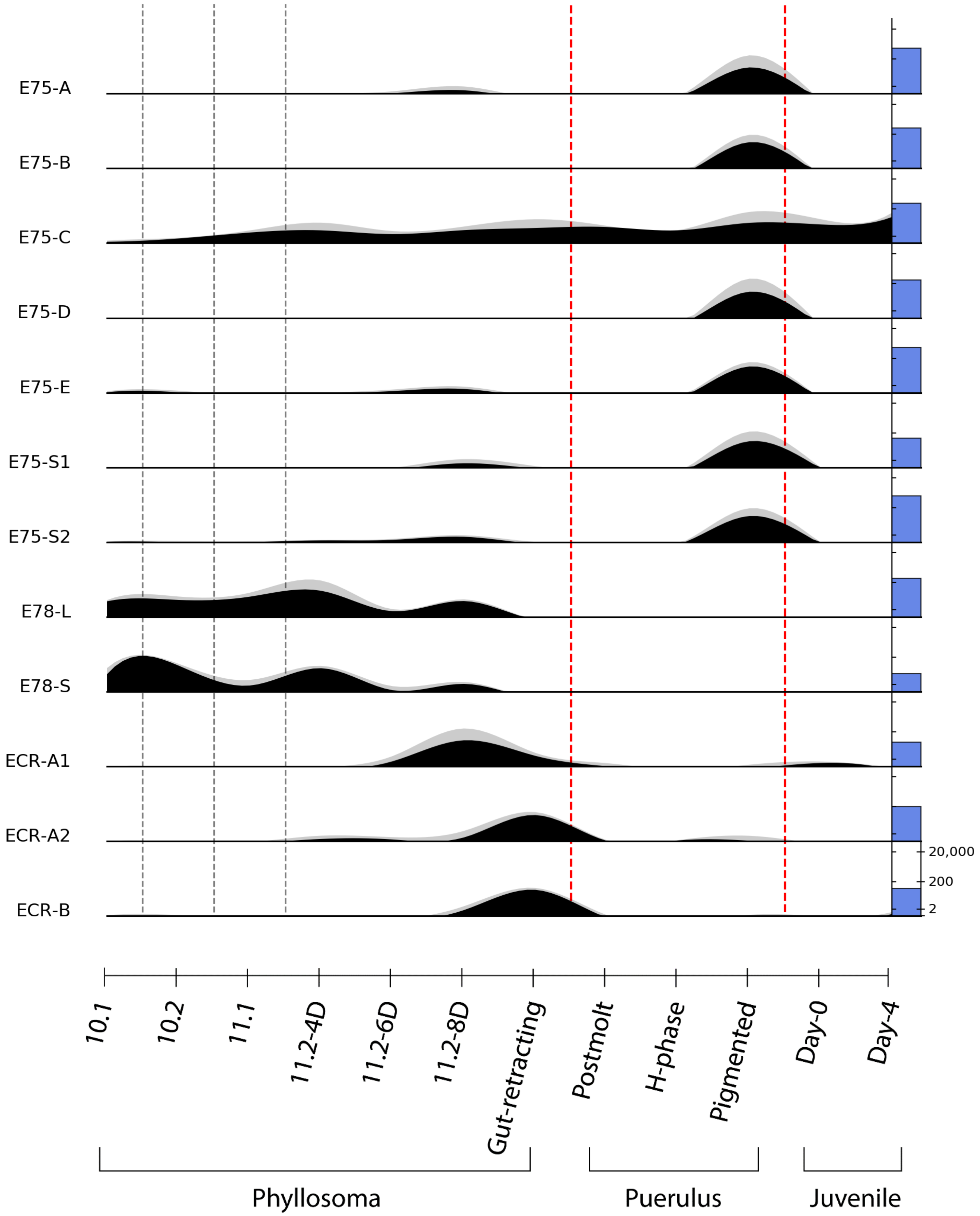

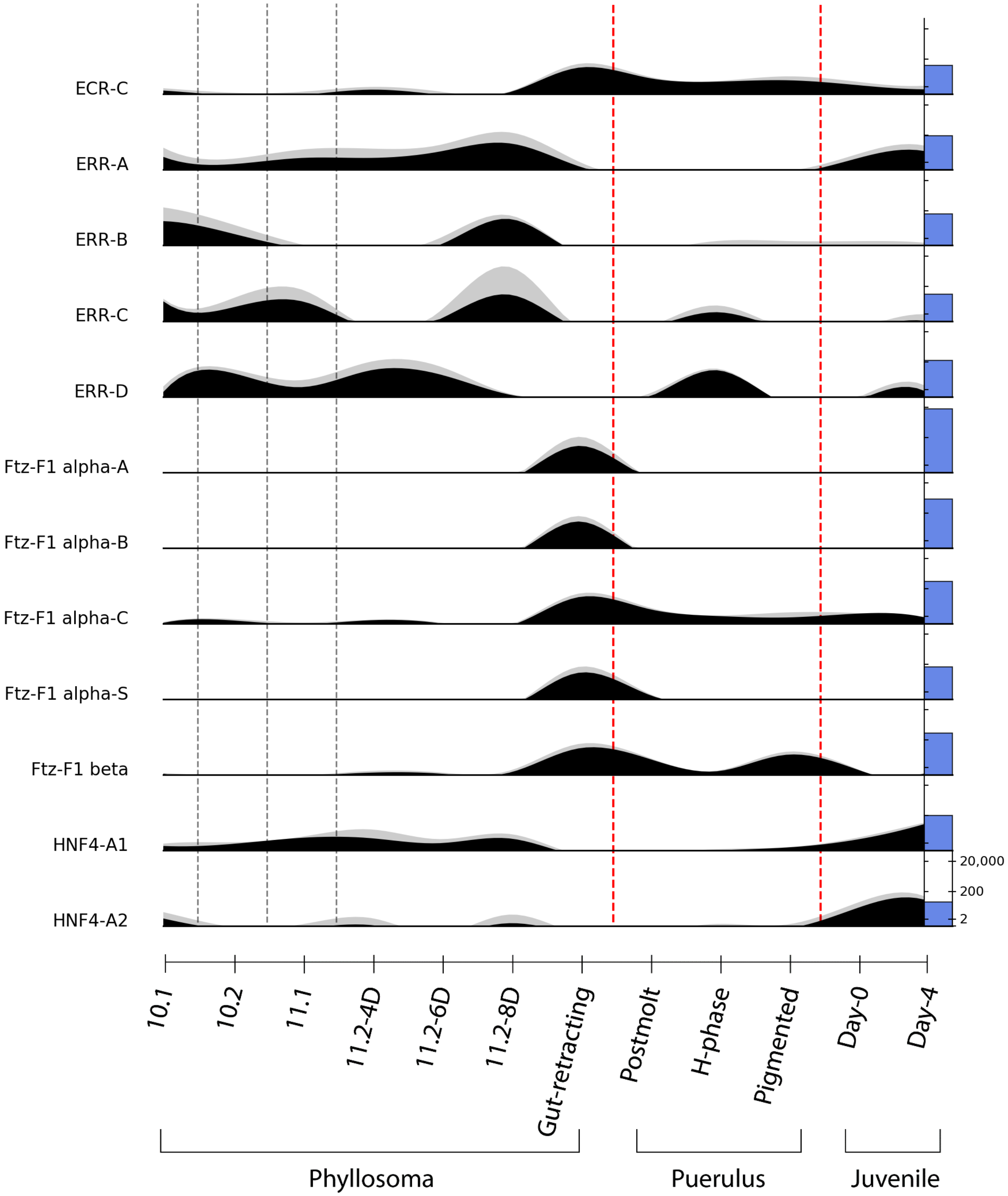

ECR-C
ERR-A
ERR-B
ERR-C
ERR-D
Ftz-F1 alpha-A
Ftz-F1 alpha-B
Ftz-F1 alpha-C
Ftz-F1 alpha-S
Ftz-F1 beta
HNF4-A1
HNF4-A2
20,000
200
2
10.1
10.2
11.1
11.2-4D
11.2-6D
11.2-8D
Gut-retracting
Postmolt
H-phase
Pigmented
Day-0
Day-4
Phyllosoma
Puerulus
Juvenile

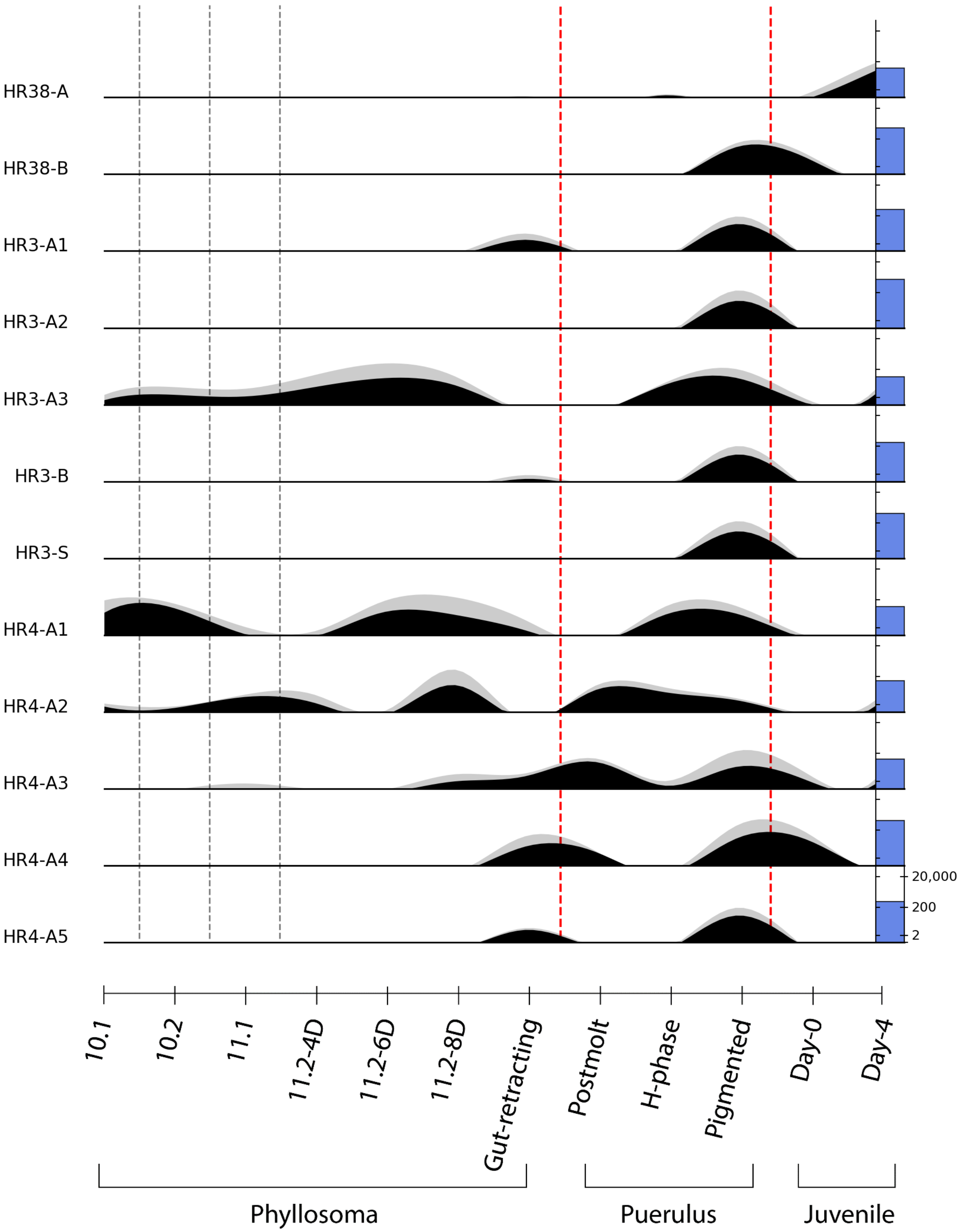
HR38-A
HR38-B
HR3-A1
HR3-A2
HR3-A3
HR3-B
HR3-S
HR4-A1
HR4-A2
HR4-A3
HR4-A4
HR4-A5
20,000
200
2
10.1
10.2
11.1
11.2-4D
11.2-6D
11.2-8D
Gut-retracting
Postmolt
H-phase
Pigmented
Day-0
Day-4
Phyllosoma
Puerulus
Juvenile

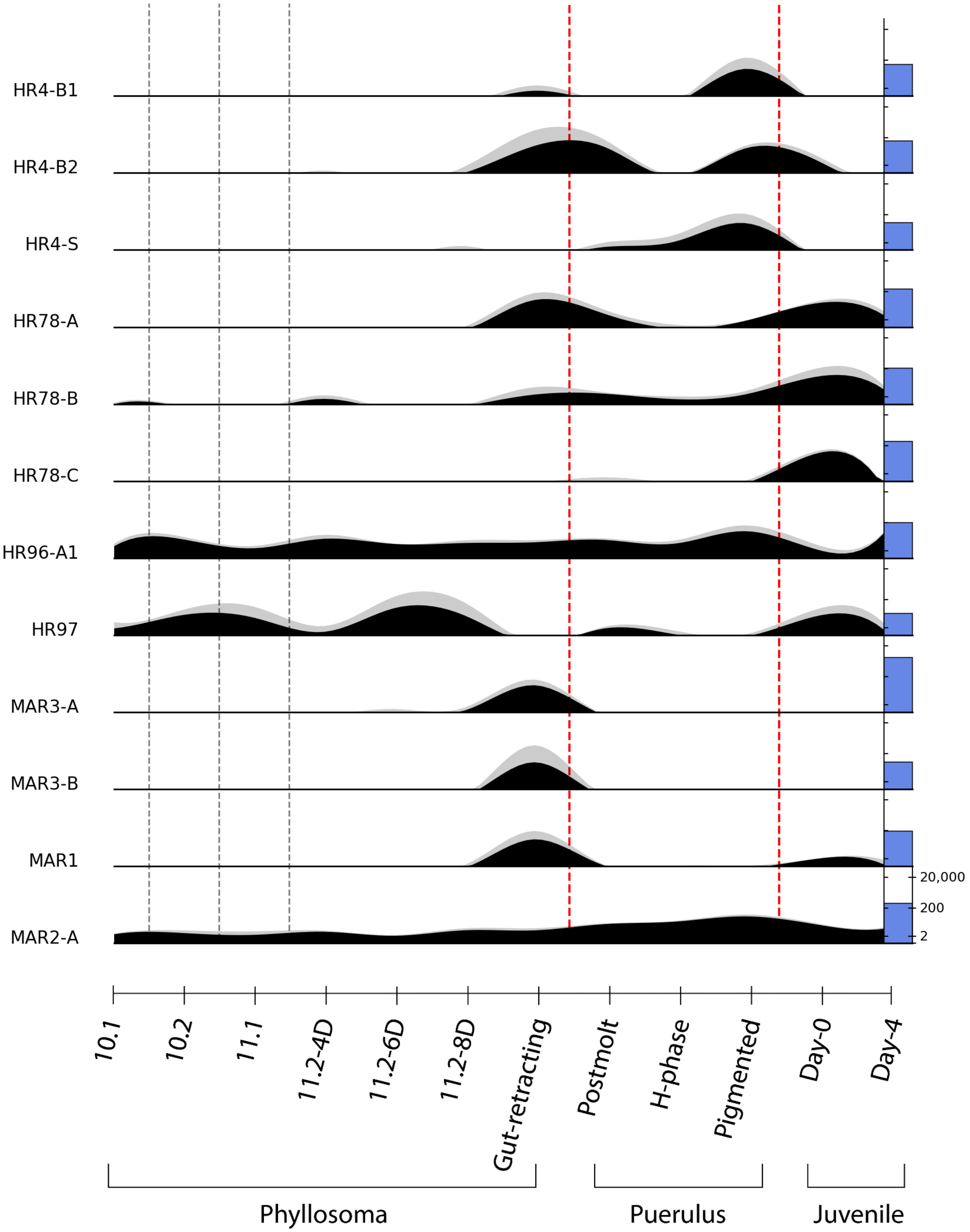

HR4-B1
HR4-B2
HR4-S
HR78-A
HR78-B
HR78-C
HR96-A1
HR97
MAR3-A
MAR3-B
MAR1
MAR2-A
20,000
200
2
10.1
10.2
11.1
11.2-4D
11.2-6D
11.2-8D
Gut-retracting
Postmolt
H-phase
Pigmented
Day-0
Day-4
Phyllosoma
Puerulus
Juvenile

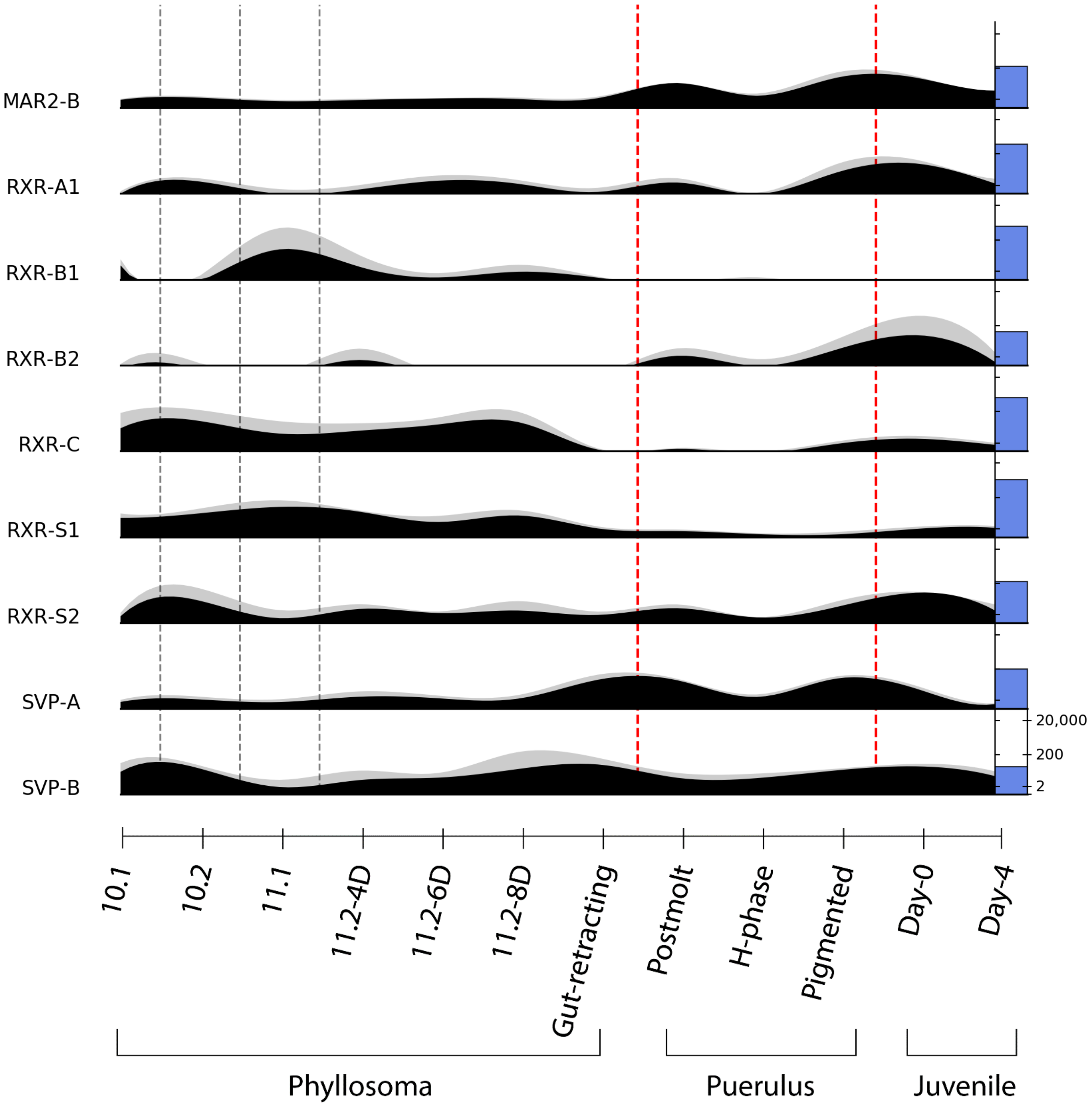
MAR2-B
RXR-A1
RXR-B1
RXR-B2
RXR-C
RXR-S1
RXR-S2
SVP-A
SVP-B
20,000
200
2
10.1
10.2
11.1
11.2-4D
11.2-6D
11.2-8D
Gut-retracting
Postmolt
H-phase
Pigmented
Day-0
Day-4
Phyllosoma
Puerulus
Juvenile
